# Attitudes About COVID-19 and Health (ATTACH): Online Survey and Mixed Methods Study

**DOI:** 10.2196/29963

**Published:** 2021-10-07

**Authors:** Anna M Hood, Hanne Stotesbury, Jennifer Murphy, Melanie Kölbel, April Slee, Charlie Springall, Matthew Paradis, Nadia Saraí Corral-Frías, Azalea Reyes-Aguilar, Alfredo B Cuellar Barboza, Amy E Noser, Stacey Gomes, Monica Mitchell, Sharon M Watkins, Melinda Butsch Kovacic, Fenella J Kirkham, Lori E Crosby

**Affiliations:** 1 Developmental Neurosciences Unit and Biomedical Research Centre University College London Great Ormond Street Institute of Child Health London United Kingdom; 2 Department of Psychology Royal Holloway University of London London United Kingdom; 3 Axio Research LLC Seattle, WA United States; 4 AirMyOpinion Ltd Weybridge United Kingdom; 5 Department of Psychology Universidad de Sonora Hermosillo, Sonora Mexico; 6 Department of Psychobiology Facultad de Psicología Universidad Nacional Autónoma de México Ciudad de México Mexico; 7 Department of Neuroscience Facultad de Psicología Universidad Nacional Autónoma de México Ciudad de México Mexico; 8 Department of Psychiatry Universidad Autonoma de Nuevo Leon Monterrey Mexico; 9 Behavioral Medicine and Clinical Psychology Cincinnati Children's Hospital Medical Center Cincinnati, OH United States; 10 College of Criminal Justice Education and Human Services University of Cincinnati College of Medicine Cincinnati, OH United States; 11 Department of Pediatrics University of Cincinnati College of Medicine Cincinnati, OH United States; 12 Cincinnati-Hamilton County Community Action Agency Cincinnati, OH United States; 13 James M Anderson Center for Health Systems Excellence Cincinnati Children’s Hospital Medical Center Cincinnati, OH United States

**Keywords:** COVID-19, mental health, international, mitigation strategies, deprivation

## Abstract

**Background:**

Behavioral mitigation strategies to slow the spread of COVID-19 have resulted in sweeping lifestyle changes, with short- and long-term psychological, well-being, and quality of life implications. The Attitudes About COVID-19 and Health (ATTACH) study focuses on understanding attitudes and beliefs while considering the impact on mental and physical health and the influence of broader demographic and geographic factors on attitudes, beliefs, and mental health burden.

**Objective:**

In this assessment of our first wave of data collection, we provide baseline cohort description of the ATTACH study participants in the United Kingdom, the United States, and Mexico. Additionally, we assess responses to daily poll questions related to COVID-19 and conduct a cross-sectional analysis of baseline assessments collected in the UK between June 26 and October 31, 2020.

**Methods:**

The ATTACH study uses smartphone app technology and online survey data collection. Participants completed poll questions related to COVID-19 2 times daily and a monthly survey assessing mental health, social isolation, physical health, and quality of life. Poll question responses were graphed using 95% Clopper–Pearson (exact) tests with 95% CIs. Pearson correlations, hierarchical linear regression analyses, and generalized linear models assessed relationships, predictors of self-reported outcomes, and group differences, respectively.

**Results:**

By October 31, 2020, 1405, 80, and 90 participants had consented to participate in the UK, United States, and Mexico, respectively. Descriptive data for the UK daily poll questions indicated that participants generally followed social distancing measures, but worry and negative impacts on families increased as the pandemic progressed. Although participants generally reported feeling that the reasons for current measures had been made clear, there was low trust that the government was doing everything in its power to meet public needs. In the UK, 1282 participants also completed a monthly survey (94.99% [1326/1396] White, 72.22% [1014/1404] female, and 20.12% [277/1377] key or essential workers); 18.88% (242/1282) of UK participants reported a preexisting mental health disorder, 31.36% (402/1282) reported a preexisting chronic medical illness, and 35.11% (493/1404) were aged over 65; 57.72% (740/1282) of participants reported being more sedentary since the pandemic began, and 41.89% (537/1282) reported reduced access to medical care. Those with poorer mental health outcomes lived in more deprived neighborhoods, in larger households (*P*s<.05), had more preexisting mental health disorders and medical conditions, and were younger than 65 years (all *P*s<.001).

**Conclusions:**

Communities who have been exposed to additional harm during the COVID-19 pandemic were experiencing worse mental outcomes. Factors including having a medical condition, or living in a deprived neighborhood or larger household were associated with heightened risk. Future longitudinal studies should investigate the link between COVID-19 exposure, mental health, and sociodemographic and residential characteristics.

## Introduction

### Background

The COVID-19 (SARS-CoV-2 virus) pandemic has placed an overwhelming burden on health systems and public health authorities to respond with effective interventions, policies, and messages [1]. Efforts to develop vaccines began quickly, with the first human clinical trial of a COVID-19 vaccine commencing on March 3, 2020, in the United States [2]. In the UK, the COVID-19 vaccine was authorized for clinical use on December 2, 2020 [3], with Mexico following soon afterward on December 11, 2020 [4]. Even with these pharmacological measures, *behavioral* mitigation strategies (eg, physical distancing, handwashing, face masks) [5] remain critical to slow the spread of COVID-19 [6]. However, the effectiveness of these behavioral strategies is dependent on adherence to policies and guidelines and on a person’s ability to perceive risks associated with the virus and adapt accordingly [7].

As the mitigation guidelines change over time and differ between countries and regions, there are many areas of uncertainty, including financial and health concerns, employment, and housing, along with fear about the future and social isolation. These sources of uncertainty may impact coping and increase the risk of developing mental health problems, with implications for quality of life in both the short and long term [8,9]. Families have had to juggle home-schooling children with working remotely or being unable to work at all [10]. Evidence from previous viral disease outbreaks indicates that when the number of stressors is high, there can be a negative effect on mental health, particularly for high-risk persons (eg, survivors and frontline health care workers) [11-15]. Currently, individuals are reporting widespread concerns about the effect of social distancing on well-being. There is evidence of increased anxiety, depression, and stress, along with reports of concern about the practical implications of the pandemic response, including for personal finances [9,16].

Social and medical factors play a significant role in COVID-19 exposure and influence the impact on mental health. It has quickly become apparent that these social and medical consequences of COVID-19 do not affect all people equally. Older adults, people with medical conditions (eg, asthma, sickle cell disease), and those facing long-standing societal inequities (ie, Black, Latinx, Indigenous, Asian, and traveler communities) face a disproportionate burden [17]. A report from Public Health England indicated that socioeconomic disadvantage, population density, and household composition may increase the likelihood not only of COVID-19 illness and severe disease [18] but also of poorer mental health outcomes [19,20]. Individuals’ concerns about their mental health outcomes will likely result in additional pressure on referral systems. Known barriers to accessing mental health interventions may also increase during the pandemic, such as distance, work commitments, and caring responsibilities.

### Goal of This Study

**Figure 1:**
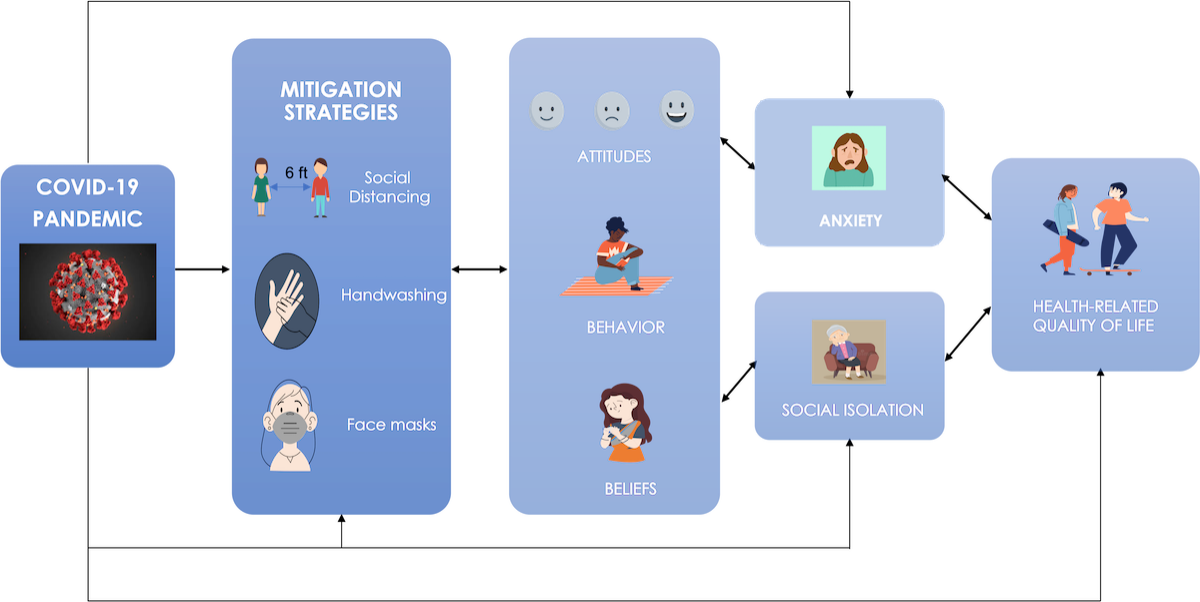
Theoretical model.

The Attitudes About COVID-19 and Health (ATTACH) study aims to understand attitudes and beliefs about the COVID-19 pandemic while considering the impact on mental and physical health, along with the influence of demographic and geographic factors. We examine how the pandemic and behavioral mitigation strategies influence attitudes and beliefs, which in turn are predicted to affect mental and physical health (eg, anxiety, social isolation) and therefore influence overall quality of life (see Figure 1 for the theoretical model). Specifically, to assess the impact of COVID-19–related measures (eg, isolation, physical distancing), our study: (1) tracks attitudes and behaviors daily as the pandemic evolves, and (2) longitudinally monitors mental and physical health symptoms using established measures that are reliable and sensitive to change.

The ATTACH study uses a smartphone app and online survey data collection. Our goals necessitate oversampling those communities that have been exposed to additional harm during the COVID-19 pandemic to guide the development of effective, and ideally, more person-centered interventions. Data encompass a sample from the United Kingdom (a high-resource country). We also include data from 2 smaller-scale studies from the United States (another high-resource country) and Mexico (a low-resource country). We chose these countries for inclusion because, before the pandemic, the Global Health Security Index ranked the United States and the UK as the first and second most prepared countries to manage a pandemic, but by June 2020, they were the first and second in excess deaths related to COVID-19 [21]. Mexico has Latin America’s second highest death toll and had 50% excess deaths when data collection began [22]. As Mexico is a primarily Spanish-speaking country with a similarly challenging COVID-19 response as the United States and the UK, the comparison could help better understand attitudes, behaviors, and mental health during the COVID-19 pandemic. Although behavioral mitigation strategies changed regularly during the study period, all 3 countries used lockdowns (eg, shelter in place), social distancing, and relied on individual compliance with the new rules. Additionally, some form of mask mandate had been introduced in all 3 countries, hospitality venues had been closed or required outdoor dining, and stores limited the number of individuals allowed inside [23-25]. However, COVID-19 vaccines had not been released in any of these countries at the time of data collection.

Data collection for ATTACH is ongoing, and longitudinal aims and specific hypotheses have been preregistered [26]. This paper primarily focuses on a cross-sectional analysis of baseline assessments collected in the UK between June 26 and October 31, 2020. It also considers longitudinal changes in UK daily poll responses through the same period. Descriptive analyses for data from the United States and Mexico are also reported, but we did not conduct statistical analyses. During the first wave, the sample sizes in those countries were less than 100 participants.

### Aims

The objectives of this study were to (1) describe the baseline characteristics of participants in the ATTACH study in the UK, United States, and Mexico; and (2) describe changes in daily UK poll responses over time in relation to specific policy interventions (eg, mask mandates) and the pandemic’s trajectory (eg, a 7-day rolling average of COVID-19 cases).

### Hypotheses

We hypothesized that residential population density, socioeconomic deprivation, and household composition will predict self-reported outcomes (ie, anxiety and depressive symptoms, social isolation, physical health, quality of life) in our UK sample; and there will be baseline differences in our UK sample in self-reported outcomes between (1) participants with and without mental health disorders, (2) participants with and without medical conditions, and (3) participants under and over the age of 65.

## Methods

### Study Design

ATTACH is a prospective cohort study conducted nationwide during the COVID-19 pandemic with arms in the UK, the United States, and Mexico. Our research team developed the study between March and April 2020. Data collection began on June 26, 2020, in the UK, on July 27, 2020, in the United States, and on October 10, 2020, in Mexico. In the UK, the ATTACH study partnered with Air My Opinion (AMO), a smartphone app that enables organizations to gather longitudinal poll data to interpret trends in attitudes and beliefs.

### Population

The ATTACH study purposely targets individuals with increased susceptibility to adverse health outcomes for recruitment, focusing on 3 priority groups: (1) those with a self-reported mental health disorder, (2) those with a self-reported chronic medical condition, and (3) those over 65 years of age. Participants were at least 16 years of age (18 years in the United States and Mexico), could read in English (UK; does not have to be their first language), Spanish (Mexico), or English or Spanish (United States). Participants had to reside in the country where the study was being completed and have access to a smartphone (UK) and the internet (UK, the United States, and Mexico). Participants provided informed electronic consent before completing daily poll questions and monthly surveys. The ATTACH study received ethical approval from the University College London (UCL) Research Ethics Committee (18177/001), the Cincinnati Children’s Hospital Medical Center Institutional Review Board (No. 2020-0465), and the Universidad de Sonora Ethics Committee (CEI-UNISON 010/2020). The study is reported in accordance with the STrengthening the Reporting of OBservational studies in Epidemiology (STROBE) [27] and the Checklist for Reporting Results of Internet E-Surveys (CHERRIES) guidelines [28].

### Materials

#### Air My Opinion App (UK Data Only)

The AMO app was customized for the ATTACH study through an iterative design process between the research team and app developer. Before the study launched, the research team appraised a mock-up design, highlighted their satisfaction and dissatisfaction with the design and format, and provided feedback and suggestions for improvement. The AMO app uses the flutter framework, has embedded encryption, and is General Data Protection Regulation (GDPR) compliant. Participants could freely install the app on an internet-enabled smartphone (Google Play or Apple App Store) running Android or iOS operating systems. Responses to daily poll questions were collected via an SMS text message service-center voting platform. Some participants (n=344) had difficulty sending their first SMS text message (ie, changed the wording, did not realize it had not been sent), which included their registration data (ie, postcode). As such, the app development team made changes so that registration data were incorporated within the app instead of being included in the first SMS text message.

**Figure 2:**
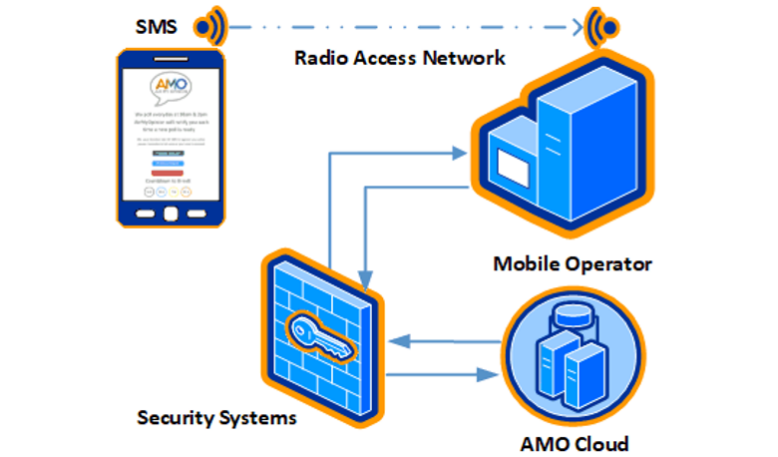
Smartphone survey polling system. AMO: Air My Opinion.

If a participant’s phone plan is the traditional pay-as-you-go, they pay for each SMS text message, but if they have a monthly plan or pay-as-you-go bundle, there is no additional cost. Each phone number is associated with a unique 1-way encrypted participant key (eg, 1::747d6f41-2f7a-47fd-a2a0-0cf41b8ca9f2 ::i2::1::484::), which feeds directly into a secure response firewall-protected database (Figure 2). According to GDPR, age range, sex, postcode, ethnic minority status (yes/no prefer not to say), and parent and chronic medical condition group status were stored in a separate secure firewall-protected database. Data protection registration has been obtained for this study (UCL Data Protection Registration Number: Z6364106/2020/04/110). The data are minimized at the first opportunity, with new keys assigned and the original keys stored separately from the rest of the data on the UCL shared drive.

#### Poll Questions (UK Data Only)

In March and April 2020, the research team conducted a literature review and held several virtual video conference meetings to develop daily poll questions using prior expertise, survey knowledge, group discussion, and reliable sources of COVID-19 information (eg, World Health Organization [WHO]). Given the fast-moving nature of the COVID-19 pandemic, validation or pilot testing of questions externally was not possible. Poll questions track attitudes, beliefs, and behaviors related to the COVID-19 pandemic and fit into broad categories related to health and well-being, personal concerns, worry or hope, compliance or rationale, government trust, and habits. All questions have 3 Likert response options (eg, yes, somewhat, not at all). There are currently 60 poll questions. New questions are added on a flexible schedule to capture changes during the pandemic while maintaining the original questions for longitudinal assessment (see Multimedia Appendix 1 for a complete list of poll questions). Most questions are repeated every 2 weeks. In this study, 6 questions were focused on descriptive analyses (see below). These questions were chosen as they were asked continuously from the beginning of the study, represent a question from each category, and were chosen before any analyses were conducted.

- In the past week, have you followed social distancing measures?
- In the past week, have you felt that COVID-19 has had a negative impact on your family?
- In the past week, how worried have you been about the ongoing COVID-19 pandemic?
- In the past week, have you felt that the reasons for the current pandemic measures have been made clear?
- In the past week, have you trusted the government to do everything in their power to ensure that the basic needs of the public are met?
- In the past week, have you spent more time than usual using social media (eg, Facebook, WhatsApp, Instagram)?

#### Monthly Survey

Baseline sociodemographic characteristics included age, sex, relationship status, educational level, first language, household composition, and caregiver status. For race and ethnicity, participants could choose from categories (based on census data from each country) or self-identify using a free response. Mental health disorders and medical conditions were identified from free response. Mental health disorders were classified based on the *Diagnostic and Statistical Manual of Mental Disorders*, 5th Edition [29] categories. To capture changes as the COVID-19 pandemic progresses, participants were asked to provide their employment and keyworker status, effects on household income, sources of and trust in COVID-19–related information, and political status on a 100-point scale (“0=left” and “100=right”) at baseline and at months 7 and 12.

Measures included in the study are validated, nonvalidated (developed rapidly during the COVID-19 pandemic), and those designed by our research team. Internal consistency (ie, Cronbach α) for this study and the assessment schedule are reported in Multimedia Appendices 2 and 3. When possible, short forms of measures were used to reduce participant burden. Measures included for analyses in this study were the Patient-Reported Outcomes Measurement Information System (PROMIS) Anxiety-Adult Short Form [30], 9-item Patient Health Questionnaire (PHQ-9) [31], 10-item University of California Los Angeles Loneliness Scale (UCLA-10) [32], PROMIS Global Health [30], the PROMIS Meaning and Purpose-Short Form [30], and the Epidemic-Pandemic Impact Inventory (Physical Health questions) [33] (Multimedia Appendix 4). PROMIS measures were available in Spanish; for the other measures, a study team member (NC-F) completed the translation.

#### Residential Risk Factors (UK Data Only)

##### Overview

Geographic region, socioeconomic disadvantage, and household composition may influence an individual’s ability to follow COVID-19–related guidelines and restrictions [18]. In our study, 2 measures captured these factors: deprivation and population density. Participants provided the first 5 characters of their UK postcode (ie, postcode sector) on the AMO smartphone app. In 2016, there were 12,381 postcode sectors in the UK. Postcode sectors vary in terms of the number of dwellings but typically range between 200 and 5000 [34].

##### Socioeconomic Deprivation

The index of multiple deprivation (IMD; 2017-2020) is an official postcode-based measure of relative deprivation in England [35], Wales [36], Scotland [37], and Northern Ireland [38] and is available as open-source government data. The IMD defines deprivation to encompass income, employment, education, health, crime, barriers to housing and services, and living environment. All neighborhoods are ranked on a relative rather than absolute scale according to their deprivation level relative to other areas. All zones are grouped into 5 bands (quintiles), each containing 20% of the zone, with “1=most deprived, 5=least deprived.” In this study, each participants’ postcode sector was assessed to determine the level of deprivation. IMD quintile scores are combined from all UK countries.

##### Residential Population Density

Population density estimates for England and Wales [39], Scotland [40], and Northern Ireland [41] are produced for each country using the cohort component method and cover the “usually resident population” from 2011 Census data. In this study, each participants’ postcode sector was assessed to determine the population density (ie, number of usual residents per hectare), and population density was combined from all UK countries.

#### Household Composition

Participants responded to the question, “How many people (adults and children) live in your household?” The 5 response options were “1” through “5 or more.”

### Procedures

**Figure 3:**
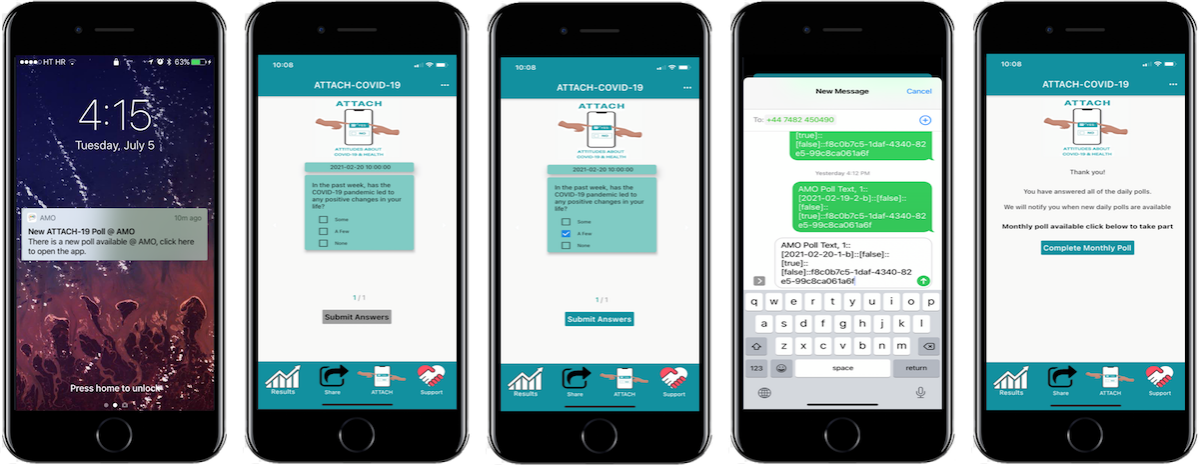
Schematic of an Attitudes About COVID-19 and Health study notification and poll question on the Air My Opinion smartphone app.

Recruitment strategies for all countries are described in Multimedia Appendix 5. Participants downloaded the smartphone app to complete consent in the UK and then provided their demographics (in case a participant chose not to complete monthly surveys) and postcode. Participants received push notifications on their smartphones to complete daily 1-minute polls 2 times each day at 10 am and 2 pm. Participants could answer both questions at the same time, but each question was removed after 24 hours. Once the participant completed the daily poll questions, they could then click on an embedded link (with a unique access code) in the app to complete their monthly survey via Research Electronic Data Capture tools (REDCap) hosted at UCL (see Figure 3 for a schematic) [42,43].

If responses to survey questions indicated that participants had severe depression or experienced high levels of stigma due to their medical condition, a pop-up message provided mental health resources along with the study email address, which was monitored regularly by a clinical psychologist (AH) to provide additional resources or referrals. After the survey, participants were redirected to the study webpages (located on the UCL Child Health Institute website), which contain links to mental health (eg, Mind), COVID-19 (eg, government), and authoritative medical information (eg, National Health Service [NHS]). Participants could see the results of daily poll questions (as pie charts) by clicking on a link in the app or via the study Twitter account.

In the United States, participants completed poll questions and monthly surveys using REDCap, and in Mexico, participants completed monthly surveys using Qualtrics (Qualtrics International Inc) with open survey links. Participants in the UK and United States who completed the monthly survey were entered into a monthly prize draw (UK £10 [US $13.75]/US $10 Amazon card). In Mexico, most participants did not receive any compensation. A subset of the sample (ie, undergraduate students) had the opportunity to receive *culture credits* (obtained through research studies or going to concerts or workshops) for their participation.

### Statistical Analysis

Analyses were conducted using R version 4.0.3 [44] and SAS version 9.4 [45]. Descriptive statistics summarized demographic and clinical characteristics. The PROMIS [30] Anxiety, Physical Health, and the Meaning and Purpose scales were scored using the online HealthMeasures scoring service [46] that utilizes information from each item to calculate a T-score (mean 50 [SD 10]). For the PHQ-9 and UCLA-10 scales, raw scores were calculated in line with published norms. Mean imputation was used when a participant completed at least 80% of items (ie, 8/10 for the UCLA-10 or 8/9 for the PHQ-9) [47,48]. The frequency of *somewhat* and *yes* poll question responses from July 15, 2020, to October 15, 2020, was graphed based on the timing of response using 95% Clopper–Pearson (exact) tests with 95% CIs, alongside the 7-day average counts of daily COVID-19 cases (per 100 persons) and deaths. Pearson correlations examined the relationship between household composition and self-reported outcomes. Hierarchical linear regression analyses assessed whether residential socioeconomic deprivation and population density independently predicted self-reported outcomes after controlling for age and sex.

Generalized linear models were used to produce adjusted least-square mean scores and differences to compare groups (participants with and without mental health and medical conditions, those under and over 65 years). Analyses controlled for demographic factors (ie, age, sex, level of residential socioeconomic deprivation) and comorbidities (ie, mental health and medical condition). All analyses were conducted using pairwise deletion, as variables generally contained less than 1% of missing data. Adjusted *P* values were based on the model *t*-statistics. Semipartial η^2^ was used as a measure of effect size to describe the proportion of total variation accounted for by the effect being tested. Statistical significance was determined at an α level of *P*<.05 (2 tailed).

### Data Exclusion

The smartphone app and online survey data were examined for duplicates by matching unique app identifiers and participant numbers. Through this process, it was determined that 15 participants had downloaded the app more than once. Duplicate data were excluded before analyses.

### Power

The ATTACH study was powered to detect longitudinal effects and not baseline group differences [26], so power calculations were not performed for these largely descriptive first-wave baseline assessments. However, for our longitudinal analyses with 3 subgroups, 5 covariates, 5 or more repeated measures at 0.80 power, an error probability of 0.05, and an effect size of 0.3, the sample size required for between-participants analyses is 153 and 50 for within-participants analyses.

### Data Sharing

Data will be shared upon reasonable request and with permission according to the ATTACH Group data release policy.

## Results

### UK Preliminary Analyses

By October 31, 2020, 1405 individuals had downloaded the smartphone app and consented to participate in the UK ATTACH study. As of October 31, 2020, the study link that takes participants to the study recruitment page had been clicked on 5068 times, with Facebook and Twitter being the most common referrers. No participants reached out via email or social media to indicate that they had a mental health concern.

A total of 123/1405 participants (8.75%) answered poll questions on at least one occasion but did not complete the monthly survey. Sensitivity analyses indicated that participants who did and did not go on to complete the survey were similar in age (*P*=.24), sex (*P*=.62), level of deprivation (*P*=.83), and UK country of residence (*P*=.74). However, those who did not complete a monthly survey were significantly more likely to identify as non-White (*P*<.001) and were from a more populated geographic region (*P*=.02) than those who did. Analyses assessed whether there were differences between participants who did and did not (n=344) send their first SMS text message correctly. They did not differ in terms of identified race (*P*=.54), but they were significantly older (*P*<.001) and more likely to be female (*P*=.02).

### UK Poll Questions

**Figure 4:**
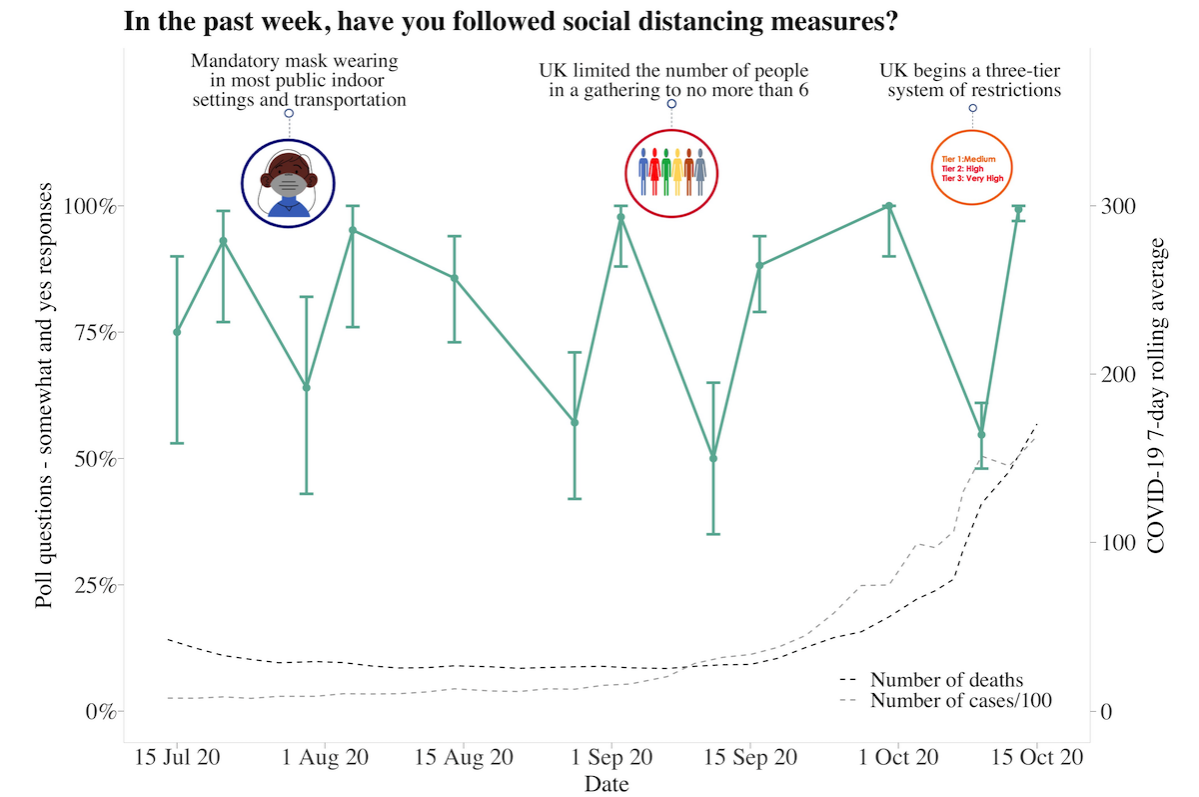
Graphs represent United Kingdom longitudinal daily poll responses for COVID-19-related question in the category of health from July 15, 2020, to October 15, 2020. Error bars represent 95% CIs. Participant responses “somewhat” and “yes” are grouped together for analyses.

**Figure 5:**
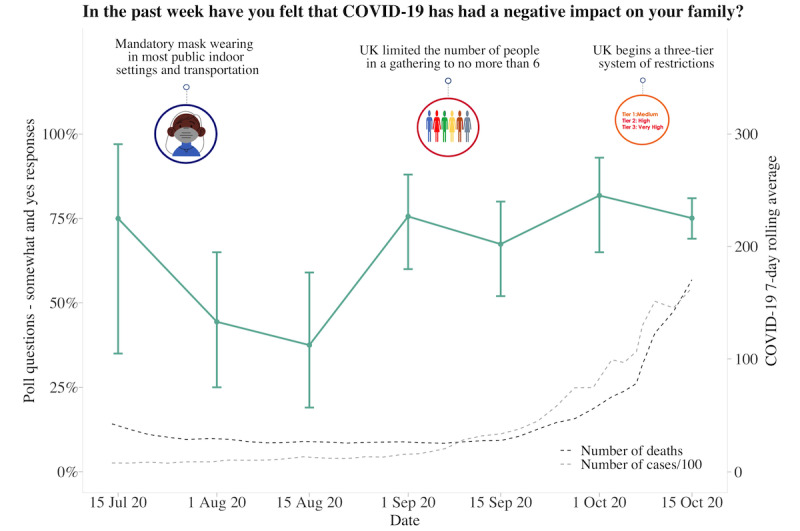
Graphs represent United Kingdom longitudinal daily poll responses for COVID-19-related question in the category of personal concerns from July 15, 2020, to October 15, 2020. Error bars represent 95% CIs. Participant responses “somewhat” and “yes” are grouped together for analyses.

**Figure 6:**
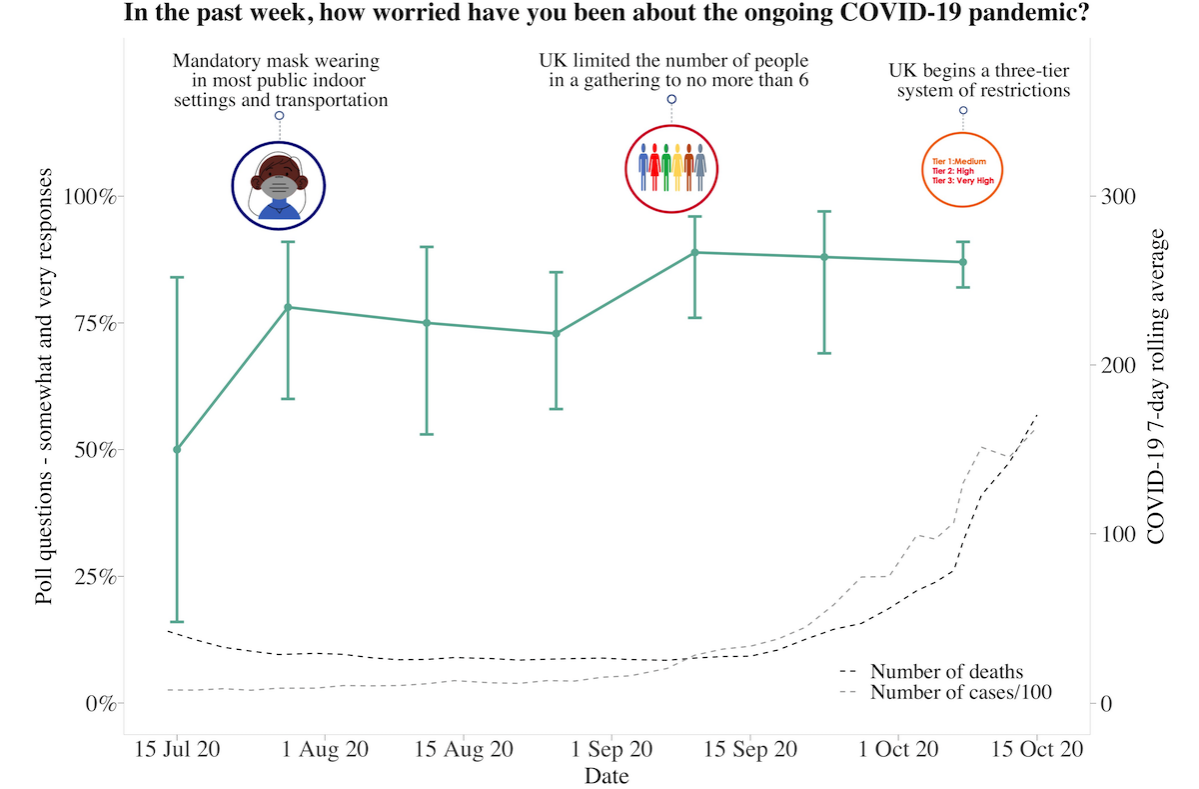
Graphs represent United Kingdom longitudinal daily poll responses for COVID-19-related question in the category of worry or hope from July 15, 2020, to October 15, 2020. Error bars represent 95% CIs. Participant responses “somewhat” and “yes” are grouped together for analyses.

**Figure 7:**
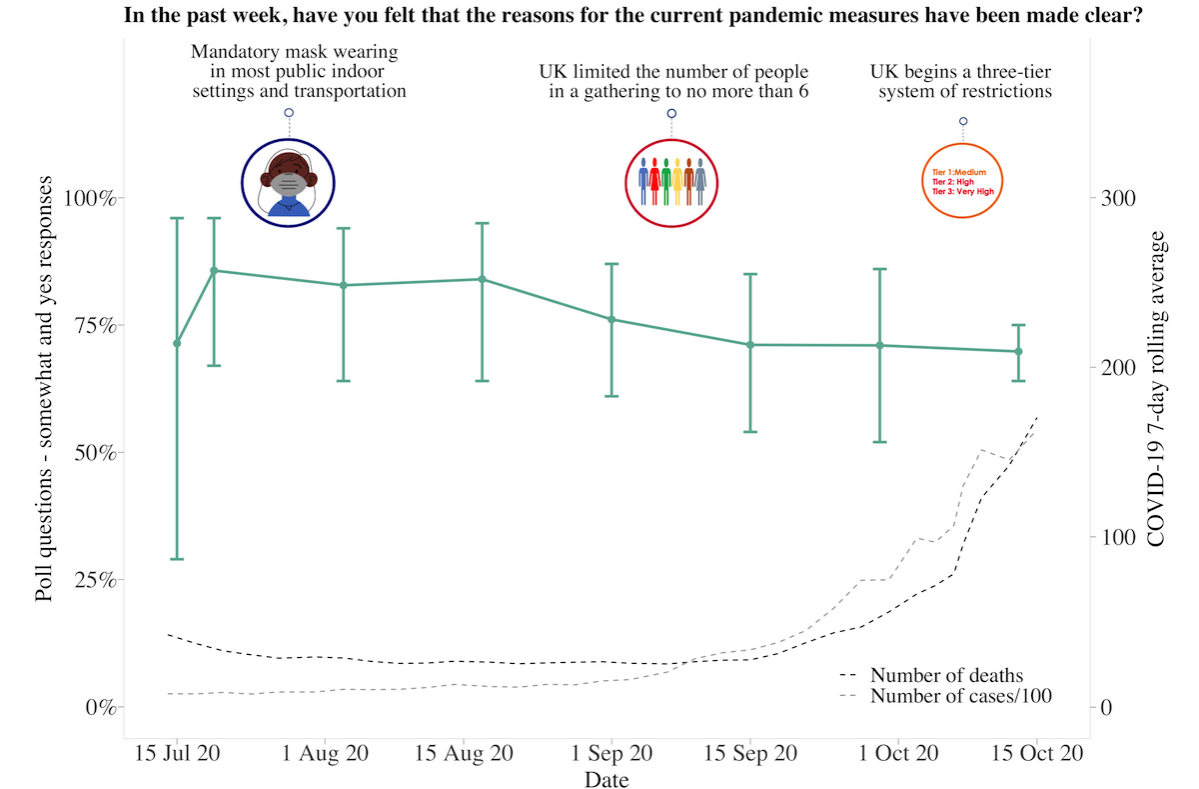
Graphs represent United Kingdom longitudinal daily poll responses for COVID-19-related question in the category of compliance or rationale from July 15, 2020, to October 15, 2020. Error bars represent 95% CIs. Participant responses “somewhat” and “yes” are grouped together for analyses.

**Figure 8:**
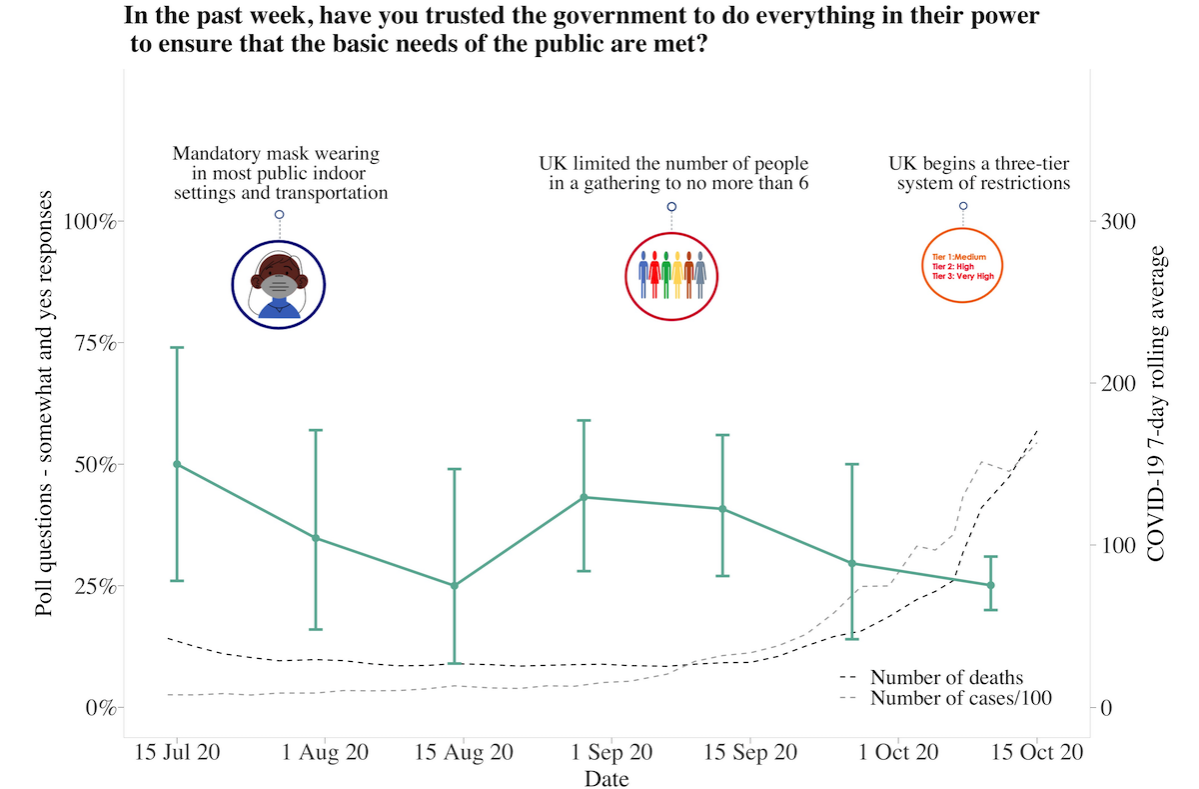
Graphs represent United Kingdom longitudinal daily poll responses for COVID-19-related question in the category of government trust from July 15, 2020, to October 15, 2020. Error bars represent 95% CIs. Participant responses “somewhat” and “yes” are grouped together for analyses.

**Figure 9:**
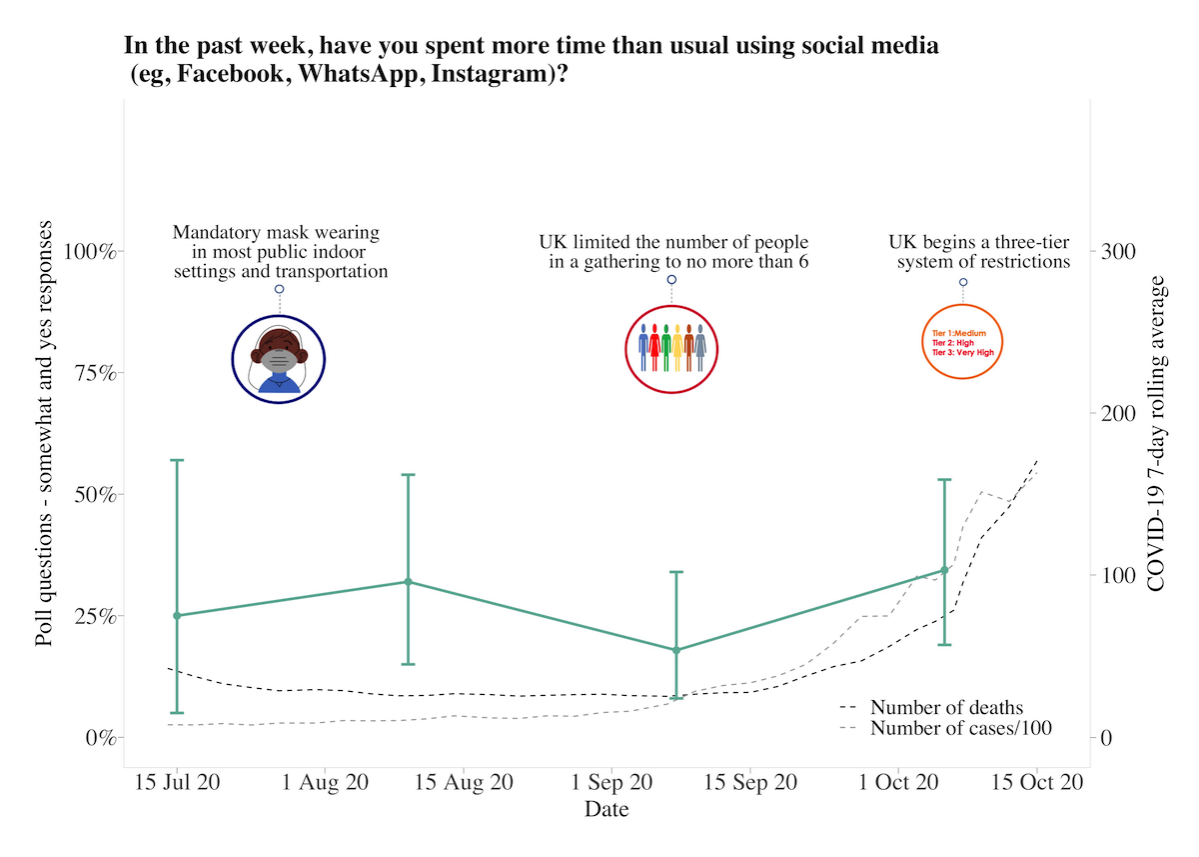
Graphs represent United Kingdom longitudinal daily poll responses for COVID-19-related question in the category of habits from July 15, 2020, to October 15, 2020. Error bars represent 95% CIs. Participant responses “somewhat” and “yes” are grouped together for analyses.

About 62.63% (880/1405) of participants answered each poll question separately at 10 am and 2 pm, with the fastest response at 8 seconds and the slowest response at 23 hours and 58 minutes. The average time to answer 1 question was 2 hours and 17 minutes, with 11.88% (167/1405) of participants answering within 10 minutes. Longitudinal data from 6 poll questions indicated that participants had generally followed social distancing measures, although there was variability across time (responses varied between 50% and 99%). Regarding whether the COVID-19 pandemic had a negative impact on the family and whether participants were worried about the pandemic, both increased over time. Over 80% of participants responded “somewhat or yes” as the pandemic progressed and as the 7-day rolling average of cases and deaths increased. Although participants generally reported feeling that the reasons for current measures had been made clear (70%-85% across the study period), there was much less trust that the government was doing everything in their power to meet public needs, with less variability in responses over time (25%-50% across the study period). Increased social media use remained consistent, with 30%-40% of participants reporting spending more time than usual (Figures 4-9).

### UK Monthly Survey

#### Descriptive Data

Participants at baseline had a mean age of 57 years, with the majority identifying as White (1326/1396, 94.99%), female (1014/1404, 72.22%), married (765/1282, 59.67%), speaking English as a first language (1231/1282, 96.02%), and educated to the college/university level or higher (996/1282, 77.69%). A total of 540/1262 participants (42.78%) were retired, and about one-fifth (277/1377, 20.12%) were key or essential workers. Most lived in a 2-person household (681/1282, 53.12%), and a minority were parents of children under 16 years (139/1282, 10.84%). Less than a quarter (300/1259, 23.83%) reported that their income had been affected by the COVID-19 pandemic, and when reporting where they see themselves on the political spectrum, scores ranged from 0 to 100 (mean 42.3 [SD 22.2]; Multimedia Appendix 6).

A subgroup of participants reported preexisting mental health disorders (242/1282, 18.88%), with depression and anxiety disorders being the most commonly reported. Just over one-third (402/1282, 31.36%) reported a preexisting chronic medical illness, with asthma, Type 1 and 2 diabetes, and arthritis being the most commonly reported (Multimedia Appendix 7). Some participants also had comorbid (ie, more than 1) mental health disorders (94/1282, 7.33%) or chronic medical illnesses (71/1282, 5.54%). Additionally, some participants had at least one mental health disorder *and* at least one chronic medical condition (100/1282, 7.80%; Multimedia Appendix 8). Scores on self-reported outcome measures indicated that just under one-third of the sample (352/1244, 28.30%) was experiencing moderate-to-severe anxiety symptoms, while just under one-fifth (233/1217, 19.15%) was experiencing moderate-to-severe depressive symptoms. Most of the sample (985/1232, 79.95%) reported feeling some loneliness or social isolation, while 248/1208 (20.53%) participants reported poor-to-fair physical health, and 476/1206 (39.47%) reported poor-to-fair quality of life (Multimedia Appendix 8).

#### Impact of the COVID-19 Pandemic

Participants reported how the COVID-19 pandemic had changed their lives and impacted their household members. Generally, fewer participants reported physical health changes, although many had received less routine or preventative medical care. At least one-third of the sample reported being less physically active and eating less healthy foods. In fact, 57.72% (740/1282) of participants reported being more sedentary since the pandemic began (Table 1).

**Table 1:** COVID-19 pandemic–related changes for participants and people in their home at baseline in the UK ATTACH study from June 26 to October 31, 2020.

COVID-19 pandemic–related changes	Participant, %^a^	Person in home, %^a^
Spent more time sitting down or being sedentary	57.7	30.8
Got less medical care than usual (eg, routine or preventive care appointments)	41.7	23.0
Less physical activity or exercise	40.7	23.7
Overeating or eating more unhealthy foods (eg, junk food)	31.9	16.8
Increase in health problems not related to this disease	21.1	12.3
Important medical procedure cancelled (eg, surgery)	6.9	5.4
Elderly or disabled family member not in the home/unable to get the help they need	5.5	6.9
Unable to access medical care for a serious condition (eg, dialysis, chemotherapy)	2.3	1.3

^a^n is not reported because it is different for each question as not all participants answered each question. The n ranged from 1280 to 1282 participant responses for each question.

#### Residential Risk Factors

Pearson correlations demonstrated that residential socioeconomic deprivation was significantly related to increased population density (*r*=–0.24; *P*<.001) and household composition (*r*=0.07; *P*=.04). Higher household composition (ie, more people residing in the home) was, however, related to *less* residential population density (*r*=–0.06; *P*=.09), although this result did not reach significance.

#### Socioeconomic Deprivation

Younger age, female sex, and living in a more deprived neighborhood (ie, quintile 1 vs quintiles 2 through 5) were predictive of reporting more anxiety symptoms, *F*_8,880_=23.12, *r*^2^=0.13 (95% CI 0.09-0.17; *P*<.001). For our second analysis, younger age and living in a more deprived neighborhood (ie, quintile 1 vs quintiles 2 through 5), but not sex, were predictive of reporting more depressive symptoms, *F*_6,862_=25.87, *r*^2^=0.15 (95% CI 0.11-0.19; *P*<.001). For our third and fourth analyses, age (*P*=.40 and .17, respectively) and sex (*P*=.22 and .34, respectively) were not significant predictors, but living in a more deprived neighborhood (ie, quintile 1) compared with living in a less deprived neighborhood (quintiles 2 through 5) was predictive of reporting worse physical health, *F*_6,860_=2.88, *r*^2^=0.13 (95% CI 0.00-0.04; *P*<.001) and more social isolation (only the least deprived quintile), *F*_6,872_=11.60, *r*^2^=0.08 (95% CI 0.04-0.10; *P*<.001). For our fifth analysis on quality of life, the overall model was significant, *F*_6,860_=6.19, *r*^2^=0.03 (95% CI 0.01-0.06; *P*<.001). However, only female sex (*P*=.03) was a significant predictor of worse quality of life. Overall, these analyses indicated that living in the most deprived neighborhoods (quintile=1) predicted worse mental health, increased social isolation, and poorer physical health.

#### Residential Population Density

Hierarchical linear regression analyses assessed whether residential population density significantly predicted self-reported outcomes after controlling for the age and sex of participants. We found that age (*P*s<.01) but not sex (*P*s>.05) was a significant predictor in all 5 regression models, indicating that younger participants were more likely to live in neighborhoods with higher population density. With regard to self-reported outcomes, the severity of anxiety and depressive symptoms and reporting more social isolation and poorer physical health were not predictive of living in a more populated neighborhood (*P*s>.05). By contrast, participants who reported poor quality of life were more likely to live in a more populated area than those who reported excellent quality of life, t_861_=2.21, β=–.31 (95% CI –0.58 to –0.03; *P*=.03).

#### Household Composition

Pearson correlations found small, but significant relationships between higher household composition and more anxiety (*r*=0.07; *P*=.01) and depressive symptoms (*r*=0.07; *P*=.01). Higher household composition was, however, related to *less* social isolation (*r*=–0.06; *P*=.07), although this result did not reach significance. Household composition was not related to physical health or quality of life (*P*s>.05).

#### Differences Between Groups

We found that participants with mental health disorders reported significantly more anxiety and depressive symptoms, more social isolation, worse physical health, and poorer quality of life than those without a mental health disorder (all *P*s<.001) after controlling for age, sex, level of residential socioeconomic deprivation, and comorbid medical conditions (Table 2). Tables 2-4 focus on specific group contrasts; however, the covariates in each model are identical. Thus, the following model-level goodness-of-fit statistics apply to each model: Anxiety (PROMIS 7 T-score), R^2^=0.224; Depression (PHQ-9 total score), R^2^=0.298; Social Isolation (UCLA 10-item total), R^2^=0.114; Social Isolation (UCLA 3-item total), R^2^=0.111; Physical Health (PROMIS T-score), R^2^=0.204; and quality of life (PROMIS T-score), R^2^=0.091.

After controlling for age, sex, level of residential socioeconomic deprivation, and comorbid mental health disorders, we found that participants with medical conditions reported significantly more anxiety (*P*=.003) and depressive symptoms (*P*=.002), and worse physical health (all *P*<.001) than those without a medical condition. Social isolation and quality of life were similar for both groups (*P*s>.05; Table 3).

After controlling for sex, level of residential socioeconomic deprivation, and comorbid mental health *and* medical conditions, we found that contrary to hypotheses, participants under 65 years of age reported significantly more anxiety and depressive symptoms and more social isolation than those over 65 years of age (all *P*s<.001). They also reported slightly better quality of life (*P*=.05), although both groups were in the average range according to the measure classifications. Participants over and under 65 years reported similar physical health (*P*=.88; Table 4).

**Table 2:** Differences in self-reported outcomes for those with and without mental health disorders.

Self-reported outcomes	n	No mental health disorder, LSM^a^ (SE)	n	Mental health disorder, LSM (SE)^b^	LSM difference (CI)	*t* (*df*)	η^2^	*P*
Anxiety: PROMIS 7a^c^ T-score	987	52.2 (0.4)	227	60.6 (0.7)	–8.4 (–9.8 to –6.9)	–11.3 (866)	0.9	<.001
Depression: PHQ-9^d^ total score	970	4.0 (0.2)	218	10.4 (0.4)	–6.4 (–7.2 to –5.6)	–15.5 (849)	0.6	<.001
Social Isolation: UCLA^e^ 10-item total	978	19.4 (0.3)	224	23.4 (0.5)	–4.1 (–5.1 to –3.1)	–8.1 (858)	0.9	<.001
Social Isolation: UCLA 3-item total	978	6.3 (0.1)	224	7.6 (0.2)	–1.3 (–1.7 to –1.0)	–7.1 (858)	0.9	<.001
Physical Health: PROMIS T-score	963	51.7 (0.4)	217	46.3 (0.7)	5.4 (4.0 to 6.8)	7.4 (847)	1.0	<.001
QOL^f^: PROMIS T-score	961	48.9 (0.5)	217	41.6 (0.9)	7.4 (5.6 to 9.2)	8.0 (847)	1.0	<.001

^a^LSM: least square mean.

^b^SE: standard error.

^c^PROMIS: Patient-Reported Outcomes Measurement Information System.

^d^PHQ-9: 9-item Patient Health Questionnaire.

^e^UCLA: UCLA Loneliness Scale.

^f^QOL: quality of life.

**Table 3:** Differences in self-reported outcomes for those with and without medical conditions.

Self-reported outcomes	n	No medical condition, LSM^a^ (SE)	n	Medical condition, LSM (SE)^b^	LSM difference (CI)	*t* (*df*)	η^2^	*P*
Anxiety: PROMIS 7a^c^ T-score	848	56.2 (2.0)	396	58.1 (2.1)	–1.9 (–3.1 to –0.6)	–2.9 (866)	1.0	.003
Depression: PHQ-9^d^ Total score	831	8.2 (1.1)	386	9.3 (1.1)	–1.1 (–1.8 to –0.4)	–3.1 (849)	0.5	.002
Social Isolation: UCLA^e^ 10-item total	842	21.1 (1.4)	390	21.6 (1.4)	–0.5 (–1.3 to 0.4)	–1.5 (858)	0.9	.25
Social Isolation: UCLA 3-item total	842	6.7 (0.5)	390	6.9 (0.5)	–0.3 (–0.6 to 0.02)	–1.9 (858)	0.9	.06
Physical Health: PROMIS T-score	823	45.8 (2.0)	385	52.6 (2.0)	6.9 (5.6 to 8.1)	11.1 (847)	1.0	<.001
QOL^f^: PROMIS T-score	821	43.2 (2.5)	385	42.6 (2.5)	0.7 (–0.9 to 2.2)	0.8 (847)	1.0	.40

^a^LSM: least square mean.

^b^SE: standard error.

^c^PROMIS: Patient-Reported Outcomes Measurement Information System.

^d^PHQ-9: 9-item Patient Health Questionnaire.

^e^UCLA: UCLA Loneliness Scale.

^f^QOL: quality of life.

**Table 4:** Differences in self-reported outcomes for those over and under 65 years.

Self-reported outcomes	n	Under 65 years, LSM (SE)^a^	n	Over 65 years, LSM (SE)^b^	LSM difference (CI)	*t* (*df*)	η^2^	*P*
Anxiety: PROMIS 7a^c^ T-score	800	58.6 (2.0)	444	55.7 (2.1)	3.0 (1.7 to 4.2)	4.7 (866)	1.0	<.001
Depression: PHQ-9^d^ Total score	827	9.8 (1.1)	454	7.8 (1.2)	2.0 (1.3 to 2.7)	5.7 (849)	0.5	<.001
Social Isolation: UCLA^e^ 10-item total	827	22.2 (1.4)	454	20.5 (1.4)	1.7 (0.8 to 2.5)	3.9 (858)	0.9	<.001
Social Isolation: UCLA 3-item total	827	7.1 (0.5)	454	6.5 (0.5)	0.6 (0.3 to 1.0)	4.1 (858)	0.9	<.001
Physical health: PROMIS T-score	827	49.2 (2.0)	454	49.3 (1.9)	0.1 (-1.1 to 1.3)	0.2 (847)	1.0	.88
QOL^f^: PROMIS T-score	827	42.1 (2.5)	454	43.6 (2.5)	-1.5 (-3.0 to -0.1)	-1.98 (847)	1.0	.05

^a^LSM: least square mean.

^b^SE: standard error.

^c^PROMIS: Patient-Reported Outcomes Measurement Information System.

^d^PHQ-9: 9-item Patient Health Questionnaire.

^e^UCLA: UCLA Loneliness Scale.

^f^QOL: quality of life.

### The United States

At baseline, participants in the smaller-scale US ATTACH study (n=90) were younger than the UK sample (mean 47.1 [SD 13.1]), more likely to identify as Black/African American (45/90, 50%), be employed (69/90, 77%), be key or essential workers (40/90, 44%), be parents of children under 16 years (34/90, 38%), and be educated at the college/university level or higher (85/90, 94%). Participants in the United States were less likely than those living in the UK to report that they were married (42/90, 47%), have a mental health disorder (12/90, 13%), and when reporting where they see themselves on the political spectrum; scores ranged from 0 to 100 (mean 34.0 [SD 21.6]). The 2 samples were similar in that the majority were female (68/90, 76%), English speakers (78/90, 87%), reported having a medical condition (26/90, 29%), most lived in a 2-person household (33/90, 37%), and less than a quarter (22/90, 24%) reported that their income had been affected by the COVID-19 pandemic (Multimedia Appendix 9).

### Mexico

During the first wave of baseline assessments, the Mexico ATTACH study (n=80) had been collecting data for less than 1 month. Participants were more likely than the UK sample to be younger than 40 years (71/80, 89%), identify as mixed/multiple ethnic groups (40/80, 50%), be single (48/90, 53%), be unemployed or employed without income (ie, furloughed; 42/80, 53%), to live in a 3-person household or larger (58/80, 73%), and be a caregiver to a child under 16 years (17/80, 21%). Similar to the UK sample, most participants were female (59/80, 74%), were educated to the college/university level or higher (48/80, 60%), had a mental health disorder (22/80, 28%), and when reporting where they see themselves on the political spectrum; scores ranged from 0 to 100 (mean 46.9 [SD 25.3]; Multimedia Appendix 10).

## Discussion

### Principal Findings

This paper offers a description of longitudinal trends in attitudes and behaviors related to the COVID-19 pandemic. Additionally, we report descriptive data from 2 smaller-scale mirror studies conducted in the United States and Mexico for comparison. Descriptive data for the UK daily poll questions indicated that participants generally followed social distancing measures, but worry and negative impact on families increased as the pandemic progressed. Our cross-sectional baseline assessment in a UK adult population indicated that those with poorer mental health outcomes lived in more deprived neighborhoods, in larger households, had more preexisting mental health disorders and medical conditions, and were younger than 65 years.

In terms of the UK pandemic trajectory, cases and deaths were relatively stable for most of this period, with more rapid increases observed only in the final month. UK longitudinal smartphone data from 6 poll questions indicated that as the pandemic progressed and the 7-day rolling average of cases and deaths began to increase rapidly, participants became more worried about the pandemic with a corresponding negative impact on their families. Although most people felt that information about the COVID-19 pandemic had been conveyed clearly, their trust in the government response was much lower. Nevertheless, participants reported generally following social distancing measures (responses varied between 50% and 99%), and 30%-40% of participants also reported spending more time than usual using social media, even though the average age of our UK sample was nearly 60 years.

Cross-sectional data from our first-wave baseline assessments in the UK yielded several important findings. Participants with preexisting mental health disorders reported worse outcomes across all mental health and psychosocial indicators. Unsurprisingly, those with preexisting mental disorders reported more symptomatology. However, those with medical conditions and younger participants (age < 65) also had increased psychological symptomatology. Specifically, 139/396 (35.1%) and 50/386 (13.0%) of participants with chronic medical conditions reported currently experiencing moderate-to-severe anxiety and depressive symptoms, respectively. Similarly, 285/827 (34.5%) and 93/827 (11.2%) of younger participants (<65 years) reported currently experiencing moderate-to-severe anxiety and depressive symptoms, respectively.

These data parallel other UK cohort studies conducted during the COVID-19 pandemic, as we found that depressive and anxiety symptomology was higher in our UK sample relative to epidemiological data collected before the COVID-19 pandemic [49-52]. We had hypothesized that older adults (>65 years) in our sample would experience worse mental health outcomes than younger participants because older people are at higher risk for worse COVID-19–related outcomes. However, after controlling for demographic factors and comorbid mental health and medical conditions, this was not the case. Instead, our data suggest chronic medical conditions are a greater risk factor than older age for poor mental health outcomes. The relationships identified between deprivation, population density, and household composition suggest that they may be surrogates for poorer housing, overcrowding, or the need to use public transportation, which increase the risk of COVID exposure.

Consistent with other online surveys assessing mental health outcomes, women were over-represented in our study sample [51]. Although this overrepresentation limits some of our conclusions, women are experiencing a disproportionate economic and employment burden related to COVID-19, along with mothers having increased childcare responsibilities [53]. These factors could have long-term deleterious effects on mental health. As our study moves into longitudinal analysis, we can determine how mental health symptoms are related to attitudes and behaviors associated with COVID-19 and whether robust, rather than uncertain, public health measures ameliorate mental health difficulties [54]. Future interventions will need to be tailored to individual and community needs while tackling entrenched preexisting mental health inequities.

Other notable findings from this study were the relationship between mental health, psychosocial outcomes, and potential risk factors, although they likely existed before the COVID-19 pandemic. Data revealed that those with poor quality of life were more likely than those with excellent quality of life to live in more populated neighborhoods. Further, higher household composition was related to more anxiety and depressive symptoms, but appeared protective with regard to social isolation. Most prominently, living in the most socioeconomically deprived neighborhoods (quintile=1) was predictive of worse outcomes for all indicators, except quality of life. These results are concerning because they indicate the potential for an exacerbation of preexisting inequities, particularly if future interventions are not tailored with a consideration of these factors. Evidence shows that those who live in the most deprived neighborhoods are hospitalized more frequently for COVID-19 infections [55]. Our results indicate that the impact of neighborhood deprivation may not only encompass physical health but may be more wide ranging and include mental health and quality of life.

Many participants reported physical health changes since the pandemic began. Strikingly, 57.72% (740/1282) of participants reported being more sedentary, and 41.89% (537/1282) reported engaging in less physical activity. These results are in conjunction with 40.95% (525/1282) of participants reporting receiving less preventative or routine medical care. Lockdown measures have reduced physical activity opportunities, and these restrictions to limit the spread of COVID-19 infection may result in a less healthy populace [51]. Given the importance of early detection for many diseases, the impact of reduced preventative care combined with poorer engagement in health-promoting behaviors may be profound. As the risk of infection may remain high for some time, medical providers, administrators, and policymakers should continue to assess procedures to optimize care for all patients as well as identify safe avenues for increasing daily physical activity and reducing time spent sedentary [56].

### Limitations

There are limitations of the findings reported in this study. A potential limitation of our survey study is self-selection bias, as participants may have joined the study because they were particularly interested in the topic. Although we used various recruitment methods, targeted efforts to improve representation, and placed a strong emphasis on anonymity and confidentiality, our UK sample was more likely to be White and from higher educational backgrounds, which is not representative of the UK population and so limits the generalizability of our findings to non-White and lower-income communities. Efforts to engage and form partnerships have begun to increase the representativeness of our sample for future longitudinal analyses. Additionally, data from Mexico ATTACH currently include over 1200 baseline assessments, so diverse between-country analyses will be possible for future waves. However, for the current UK baseline assessment, cultural factors and behavioral strategies specific to the UK, such as strong public messaging to protect the NHS, may mean our results do not generalize outside the UK.

This study began recruiting after the first UK lockdown (March 2020), so we lack real-time data from before the COVID-19 pandemic and cannot know if symptomology has changed. Further, indicators of preexisting mental health disorders and chronic medical conditions were based on self-report rather than clinical diagnoses. Given the unprecedented nature of the pandemic, we prioritized rapid deployment and data collection and so did not pilot test poll questions. However, many of the questions mirror those of other newly developed studies [57,58]. The fast changes related to the pandemic meant that we could not consider all possible predictors before study initiation, so there are no measures related to quarantine status, fear of COVID-19, duration of exposure to COVID-19–related information, and infection risk perception. Although 87% of the UK population has access to a smartphone, just 53% of those aged 65 or older have internet access via smartphone ownership [59]. Access to digital devices and the internet are also limited among other marginalized communities. Therefore, those who are digitally excluded are likely to be underrepresented in our sample.

### Strengths

Strengths of our study include the mix of cross-sectional and longitudinal data collection methodology with the ability to assess daily attitudes and behaviors during the rapidly changing COVID-19 pandemic. Through the use of smartphone technology and mHealth, we can capture real-time responses and bridge physical distance. Participants completed most measures with little missing data, indicating that items were not burdensome. Finally, although our UK sample needs to increase representation related to ethnic and racialized identities, our Mexican sample includes a cross-section of communities. Through targeted recruitment, the United States sample includes an overrepresentation of participants who identified as Black or African American.

### Implications

Smartphone apps are increasingly recognized as potentially powerful tools for mHealth studies and interventions [60,61]. They have been utilized successfully in adult and pediatric chronic illness populations [62-65] and in ecological momentary assessment studies and interventions [66,67]. Data from the UK ATTACH study demonstrate that people are also prepared to answer daily questions and provide mental health data in a longitudinal study using smartphone technology. Optimizing digital approaches and integrating them into the public health response is possible while considering logistical and technological barriers. However, currently, there is limited evidence that mHealth apps and interventions are cost-effective or cost-saving [68,69]. This engagement increases the feasibility of undertaking potentially sensitive longitudinal research and reduces retrospective judgments that tend to be affected by recall bias [67]. Because of this engagement, moving forward, the Mexico ATTACH study will use the Telegram messaging system to collect daily poll data, as they are widely accessible with an encrypted point-to-point connection. As we move forward, it will be necessary to include participants’ input early in the research process to engender long- and short-term engagement [70].

### Conclusions

Our data indicate that those with mental health disorders and chronic medical conditions are experiencing increased anxiety and depressive symptoms. Socioeconomic deprivation also appears to be a considerable risk factor for poor mental health. Although these challenges are not new, they could become more deep-rooted and challenging to tackle in this new COVID-19 era and beyond. Along with a renewed focus on mental health, investment to increase access to preventative medical care and messaging to encourage health-promoting behaviors, including physical activity, will be critical.

## Appendix

Multimedia Appendix 1 Complete list of daily poll questions and the assessment schedule.

Multimedia Appendix 2 Validated measures and assessment schedule in the ATTACH Study.

Multimedia Appendix 3 Non-validated measures and assessment schedule in the ATTACH Study.

Multimedia Appendix 4 Measures used in the ATTACH study.

Multimedia Appendix 5 Recruitment strategies and example Facebook advertisement used in the ATTACH study.

Multimedia Appendix 6 Mental health disorders and medical conditions reported in the ATTACH study.

Multimedia Appendix 7 Participant characteristics at baseline in the UK ATTACH Study from June 26 to October 31, 2020.

Multimedia Appendix 8 Participant clinical classifications and severity on self-reported outcomes at baseline in the UK ATTACH study from June 26 to October 31, 2020.

Multimedia Appendix 9 Participant characteristics at baseline in the USA ATTACH Study from June 26 to October 31, 2020.

Multimedia Appendix 10 Participant characteristics at baseline in the Mexico ATTACH Study from October 6 to October 31, 2020.

## Abbreviations

AMO: Air My Opinion
ATTACH: Attitudes About COVID-19 and Health
GDPR: General Data Protection Regulation
NHS: National Health Service
PROMIS: Patient-Reported Outcomes Measurement Information System
PHQ-9: 9-item Patient Health Questionnaire
REDCap: Research Electronic Data Capture
STROBE: STrengthening the Reporting of OBservational studies in Epidemiology
UCL: University College London
UCLA-10: 10-item University of California Los Angeles Loneliness Scale
WHO: World Health Organization
