# Supplementary material for: Attitudes About COVID-19 and Health (ATTACH): Online Survey and Mixed Methods Study
Source: JMIR Ment Health. 2021 Oct 7;8(10):e29963. doi: 10.2196/29963 (PMC8500353; doi:10.2196/29963)
Supplement: Multimedia Appendix 1 [file mental_v8i10e29963_app1.docx]

**Daily poll questions**

After consenting to the study, all participants will answer the following questions. These sign-up questions will be asked once of new participants

1) Gender

Male

Female

2) Age range

16 – 22

23 – 40

41 – 64

65 – 74

75+

3) Postcode (This is the postcode of your primary residence/home)

4) Are you considered a key worker?

Yes/No

5) Is anyone in your household considered a key worker?

Yes/No

6) Are you a member of a racial or ethnic minority group (e.g., BAME)

Yes/No/Prefer not to say

7) Do you have a medical condition (e.g., asthma, diabetes, neurological condition) that puts you at high-risk of suffering from COVID-19 complications?

Yes/No

8) Are you a parent/guardian?

Yes/No

9) Are you a parent/guardian of a child with a medical condition (e.g., asthma, diabetes, neurological condition) that puts them at high-risk of suffering from COVID-19 complications?

Yes/No

| **Initial Questions from June 26 – Oct 1 2020** | **Ans1** | **Ans2** | **Ans3** |
| --- | --- | --- | --- |
| In the past week, have you felt that the COVID-19 pandemic could bring people together or tear people apart? | Together | Apart | Unsure |
| In the past week, have you or anyone in your household tested positive for COVID-19? | Myself | Household member | No |
| In the past week, what do you think the government has been prioritizing most in their response to the COVID-19 pandemic? | Health | Finances | Security |
| In the past week, how worried have you been about the on-going COVID-19 pandemic? | Very | Somewhat | No |
| In the past week have you felt that COVID-19 has had a negative impact on your family? | Yes | Somewhat | No |
| In the past week, have you felt that the current pandemic measures are? | Fair | Excessive | Weak |
| In the past week, have you worried that lifting social distancing measures will affect your health? | Yes | Somewhat | No |
| During the past week, have you spent more time with your family? | Yes | Somewhat | No |
| For the majority of the past week, have you worked from home? | Yes | Somewhat | No |
| In the past week, have you trusted the government to do everything in their power to ensure that the basic needs of the public are met? | Yes | No | Unsure |
| In the past week, how would you describe your mental health? | Good | Average | Poor |
| In the past week, from whom have you been most concerned about health risk? | Myself | Loved Ones | Others |
| During the past week, have you spent more time with your friends? | Yes | Somewhat | No |
| In the past week have you been more concerned than usual about your finances? | Yes | Somewhat | No |
| In the past week, have you felt that the reasons for the current pandemic measures have been made clear? | Yes | Somewhat | No |
| In the past week, have you been involved in voluntary work? | Yes | Somewhat | No |
| In the past week, have you followed social distancing measures? | Yes | Somewhat | No |
| In the past week, have you felt hopeful about the future? | Yes | Somewhat | No |
| In the past week have you felt that COVID-19 has had a negative impact on your relationship with your spouse or significant other? | Yes | Somewhat | No |
| In the past week, have you been reassured by the governments’ response? | Yes | Somewhat | No |
| In the past week, have you or anyone in your household received medical treatment for COVID-19? | Myself | Household member | No |
| During the past week, have you felt closer to your friends? | Yes | Somewhat | No |
| In the past week, how much attention have you paid to signals from your body (e.g., breathing, stomach, heart)? | A Lot | A Little | None |
| In the past week, has it been difficult to fully comply with the current pandemic measures outlined by the government? | Yes | Somewhat | No |
| In the past week, what has worried you the most about the COVID-19 pandemic? | Health | Finances | Security |
| In the past week, have you or anyone in your household experienced any COVID-19 symptoms (e.g., cough, fever, loss of smell)? | Myself | Household member | No |
| During the past week, have you felt closer to your family? | Yes | Somewhat | No |
| In the past week, have you worried that there will be another pandemic? | Yes | Somewhat | No |
| In the past week, have you spent more time than usual exercising outside? | Yes | Somewhat | No |
| In the past week, have you spent more time than usual using social media (e.g., Facebook, WhatsApp, Instagram) | Yes | Somewhat | No |
| In the past week, have you felt that the COVID-19 pandemic could bring people together or tear people apart? | Together | Apart | Unsure |
| In the past week, have you or anyone in your household tested positive for COVID-19? | Myself | Household member | No |
| In the past week, what do you think the government has been prioritizing most in their response to the COVID-19 pandemic? | Health | Finances | Security |
| In the past week, how worried have you been about the on-going COVID-19 pandemic? | Very | Somewhat | No |
| In the past week have you felt that COVID-19 has had a negative impact on your family? | Yes | Somewhat | No |
| In the past week, have you felt that the current pandemic measures are? | Fair | Excessive | Weak |
| In the past week, have you worried that lifting social distancing measures will affect your health? | Yes | Somewhat | No |
| During the past week, have you spent more time with your family? | Yes | Somewhat | No |
| For the majority of the past week, have you worked from home? | Yes | Somewhat | No |
| In the past week, have you trusted the government to do everything in their power to ensure that the basic needs of the public are met? | Yes | No | Unsure |
| In the past week, how would you describe your mental health? | Good | Average | Poor |
| In the past week, from whom have you been most concerned about health risk? | Myself | Loved Ones | Others |
| During the past week, have you spent more time with your friends? | Yes | Somewhat | No |
| In the past week have you been more concerned than usual about your finances? | Yes | Somewhat | No |
| In the past week, have you felt that the reasons for the current pandemic measures have been made clear? | Yes | Somewhat | No |
| In the past week, have you been involved in voluntary work? | Yes | Somewhat | No |
| In the past week, have you followed social distancing measures? | Yes | Somewhat | No |
| In the past week, have you felt hopeful about the future? | Yes | Somewhat | No |
| In the past week have you felt that COVID-19 has had a negative impact on your relationship with your spouse or significant other? | Yes | Somewhat | No |
| In the past week, have you been reassured by the governments’ response? | Yes | Somewhat | No |
| In the past week, have you or anyone in your household received medical treatment for COVID-19? | Myself | Household member | No |
| During the past week, have you felt closer to your friends? | Yes | Somewhat | No |
| In the past week, how much attention have you paid to signals from your body (e.g., breathing, stomach, heart)? | A Lot | A Little | None |
| In the past week, has it been difficult to fully comply with the current pandemic measures outlined by the government? | Yes | Somewhat | No |
| In the past week, what has worried you the most about the COVID-19 pandemic? | Health | Finances | Security |
| In the past week, have you or anyone in your household experienced any COVID-19 symptoms (e.g., cough, fever, loss of smell)? | Myself | Household member | No |
| During the past week, have you felt closer to your family? | Yes | Somewhat | No |
| In the past week, have you worried that there will be another pandemic? | Yes | Somewhat | No |
|  |  |  |  |
| **Questions from October 2020** | **Ans1** | **Ans2** | **Ans3** |
|  |  |  |  |
| In the past week, how much help or support have you PROVIDED to family, friends, or neighbours? | A Lot | A Little | None |
| In the past week, how much help or support have you RECEIVED from family, friends, or neighbours? | A Lot | A Little | None |
| In the past week, have most of your interactions been: | Online | In-person | A mix of both |
| In the past week, how often did you do any vigorous physical activities like heavy lifting, digging, aerobics, or fast bicycling? | Most days | Some days | None |
| In the past week, have you been able to enjoy your normal day-to-day activities? | Yes | Somewhat | No |
| In the past week, how much time have you spent outside the home? (e.g., shopping, parks, etc.) | A Lot | A Little | None |
| In the past week, how worried have you been about being infected with COVID-19? | Very | Somewhat | Not at all |
| In the past week, how hopeful have you been that the COVID-19 pandemic will end soon? | Very | Somewhat | Not at all |
| In the past week, how much difficulty have you had limiting close contact with people outside of your home? | A Lot | A Little | None at all |
| In the past week, have you been able to access all of the social or health services you needed? | Yes | No | Not required |
| In the past week, has the COVID-19 pandemic led to any positive changes in your life? | Some | A Few | None |
| In the past week, have you worried that there will be a second-wave of the COVID-19 pandemic? | Yes | Somewhat | Not at all |
| In the past month, have you had to cancel an event that was important to you? (e.g., holiday, party etc.) | Yes | No | Postponed |
| In the past month, have you been concerned about the financial stability of your living situation? | A Lot | A Little | Not at all |
| In the past week, did you wear a mask when in moderate/high risk situations (e.g., shops, public transport) | Yes | Sometimes | No |
| Do you agree with the government’s current travel restrictions? | Yes | Somewhat | No |
| Are you concerned that key workers have the PPE they need? | Yes | Somewhat | No |
| Do you think a coronavirus vaccine will be available in the next few months? | Yes | Unsure | No |
| Do you feel that in-person schooling is safe? | Yes | Somewhat | No |
| In the past week, have you been worried about the UK’s economic stability? | A Lot | A Little | Not at all |
| Do you currently think that traveling by airplane is safe? | Yes | Somewhat | No |
| In the past week, have you been worried about your parent’s or older friends’ health? | A Lot | A Little | Not at all |
| In the past week, have you been worried about your job security? | A Lot | A Little | Not at all |
| Do you think contact tracing methods are working? | Yes | Unsure | No |
| Do you currently think that traveling by airplane is safe? | Yes | Somewhat | No |
| Do you think that the current COVID-19 testing levels are adequate? | Yes | Somewhat | No |
| In the past week, have you felt that the COVID-19 pandemic could bring people together or tear people apart? | Together | Apart | Unsure |
| In the past week, have you or anyone in your household tested positive for COVID-19? | Myself | Household member | No |
| In the past week, what do you think the government has been prioritizing most in their response to the COVID-19 pandemic? | Health | Finances | Security |
| In the past week, how worried have you been about the on-going COVID-19 pandemic? | Very | Somewhat | No |
| In the past week have you felt that COVID-19 has had a negative impact on your family? | Yes | Somewhat | No |
| In the past week, have you felt that the current pandemic measures are? | Fair | Excessive | Weak |
| In the past week, have you worried that lifting social distancing measures will affect your health? | Yes | Somewhat | No |
| During the past week, have you spent more time with your family? | Yes | Somewhat | No |
| For the majority of the past week, have you worked from home? | Yes | Somewhat | No |
| In the past week, have you trusted the government to do everything in their power to ensure that the basic needs of the public are met? | Yes | No | Unsure |
| In the past week, how would you describe your mental health? | Good | Average | Poor |
| In the past week, from whom have you been most concerned about health risk? | Myself | Loved Ones | Others |
| During the past week, have you spent more time with your friends? | Yes | Somewhat | No |
| In the past week have you been more concerned than usual about your finances? | Yes | Somewhat | No |
| In the past week, have you felt that the reasons for the current pandemic measures have been made clear? | Yes | Somewhat | No |
| In the past week, have you been involved in voluntary work? | Yes | Somewhat | No |
| In the past week, have you followed social distancing measures? | Yes | Somewhat | No |
| In the past week, have you felt hopeful about the future? | Yes | Somewhat | No |
| In the past week have you felt that COVID-19 has had a negative impact on your relationship with your spouse or significant other? | Yes | Somewhat | No |
| In the past week, have you been reassured by the governments’ response? | Yes | Somewhat | No |
| In the past week, have you or anyone in your household received medical treatment for COVID-19? | Myself | Household member | No |
| During the past week, have you felt closer to your friends? | Yes | Somewhat | No |
| In the past week, how much attention have you paid to signals from your body (e.g., breathing, stomach, heart)? | A Lot | A Little | None |
| In the past week, has it been difficult to fully comply with the current pandemic measures outlined by the government? | Yes | Somewhat | No |
| In the past week, what has worried you the most about the COVID-19 pandemic? | Health | Finances | Security |
| In the past week, have you or anyone in your household experienced any COVID-19 symptoms (e.g., cough, fever, loss of smell)? | Myself | Household member | No |
| During the past week, have you felt closer to your family? | Yes | Somewhat | No |
| In the past week, have you worried that there will be another pandemic? | Yes | Somewhat | No |
| In the past week, have you spent more time than usual exercising outside? | Yes | Somewhat | No |
| In the past week, have you spent more time than usual using social media (e.g., Facebook, WhatsApp, Instagram) | Yes | Somewhat | No |
| Do you think the COVID-19 pandemic is under control? | Yes | Somewhat | No |
| In the past week, have you spent more time than usual using your mobile phone? | Yes | Somewhat | Not at all |
