# Supplementary material for: Attitudes About COVID-19 and Health (ATTACH): Online Survey and Mixed Methods Study
Source: JMIR Ment Health. 2021 Oct 7;8(10):e29963. doi: 10.2196/29963 (PMC8500353; doi:10.2196/29963)
Supplement: Multimedia Appendix 2 [file mental_v8i10e29963_app2.docx]

**Multimedia Appendix 2** Validated measures used in the ATTACH Study

| **Construct** | **Measure** | **Response Format** | **Items** | **Scoring** | $\boldsymbol{\alpha}$**^a^** | **Baseline/Month 7/ Final** | **Time 2**  **Only** | **Each Month** |
| --- | --- | --- | --- | --- | --- | --- | --- | --- |
|  |  |  |  |  |  |  |  |  |
| Anxiety | PROMIS^b^ Anxiety-Adult Short Form | 5-point Likert | 7 | T-scores  ↑ worse anxiety | UK = .95  USA = .93  MX^c^ = .90 | X^d^ | –^e^ | X^d^ |
| Depression | Patient Health Questionnaire | 4-point Likert | 9 | Raw scores  ↑ worse depression | UK = .90  USA = .88  MX = .89 | X^d^ | –^e^ | X^d^ |
| Social Isolation | UCLA Loneliness Scale | 4-point Likert | 10 | Raw scores  ↑ greater feelings of loneliness | UK = .91  USA = .89  MX = .88 | X^d^ | –^e^ | X^d^ |
| Physical Health | PROMIS® Global Health | 5-point Likert | 4 | T-scores  ↑ better physical health | UK = .79  USA = .76  MX = .64 | X^d^ | –^e^ | –^e^ |
| Quality of Life | PROMIS® Meaning and Purpose – Short Form | 5-point Likert | 4 | T-scores ↑hopefulness, optimism, and goal-directedness | UK = .94  USA = .94  MX = .89 | X^d^ | –^e^ | –^e^ |
| Resilience^g^ | Brief Resilience Scale | 5-point Likert | 6 | Raw scores  ↑ better resilience | N/A^f^ | –^e^ | –^e^ | X^d^ |
| Stigma (adult)^h^ | Chronic Illness Anticipated Stigma Scale | 5-point Likert | 12 | Raw scores  ↑ anticipated stigma | N/A^f^ | X^d^ | –^e^ | –^e^ |
| Health-related stigma^i^ | Childhood Stigma Scale | 5-point Likert | 5 | Raw scores  ↑ perception of stigma | N/A^f^ | X^d^ | –^e^ | –^e^ |
| Personality | Ten-item Personality Inventory | 7-point Likert | 10 | Scores on each dimension of the Big 5 | N/A^f^ | –^e^ | X^d^ | –^e^ |

^a^α: Cronbach’s alpha.

^b^Patient-Reported Outcomes Measurement Information System

^c^MX: Mexico.

^d^X: indicates that a measure was completed at that timepoint.

^e^–: indicates that a measure was not completed at that timepoint.

^f^N/A: not applicable or data not included in this study.

^g^BRS data collected from month 3.

^h^Measure completed by individuals living with a chronic medical condition.

^i^Measure completed if the participant is the parent of a child living with a chronic medical condition.
