# Supplementary material for: Attitudes About COVID-19 and Health (ATTACH): Online Survey and Mixed Methods Study
Source: JMIR Ment Health. 2021 Oct 7;8(10):e29963. doi: 10.2196/29963 (PMC8500353; doi:10.2196/29963)
Supplement: Multimedia Appendix 3 [file mental_v8i10e29963_app3.docx]

**Multimedia Appendix 3** Non**-**validated measures used in the ATTACH Study

| **Construct** | **Measure** | **Response Format** | **Items** | **Scoring** | $\boldsymbol{\alpha}$**^a^** | **Baseline/Month 7/ Final** | **Time 2**  **Only** | **Each Month** |
| --- | --- | --- | --- | --- | --- | --- | --- | --- |
|  | | | | | | | | |
| Demographics |  | Multiple Choice  Yes/No  Scale (0-100) |  |  | N/A^b^ | X^c^ | –^d^ | –^d^ |
| Demographics |  | Multiple Choice  Yes/No  Scale (0-100) |  |  | N/A^b^ | X^c^ | –^d^ | –^d^ |
| Impact of COVID on the individual | Epidemic-Pandemic Impact Inventory (Physical Health) | Yes, No, N/A, Person in home | 8 | % of responses | N/A^b^ | X^c^ | –^d^ | –^d^ |
| Impact of COVID on the family^e^ | COVID-19 Exposure and Family Impact Survey (CEFIS)^z^ | Exposure Yes/No  Impact  4-point Likert Distress  10-point Likert  Open-ended question | 45 | Raw scores  ↑ greater COVID-19 related impact, exposure, and distress | N/A^b^ | X^c^ | –^d^ | –^d^ |
| Acceptability, feasibility, and satisfaction^f^ | Acceptability, feasibility, satisfaction scale | 3 and 4-point Likert | 25 | Raw scores  ↑ more acceptability, feasibility, and satisfaction | N/A^b^ | –^d^ | –^d^ | X^c^ |

^a^α: Cronbach’s alpha.

^b^N/A: not applicable or data not included in this study.

^c^X: indicates that a measure was completed at that timepoint.

^d^–: indicates that a measure was not completed at that timepoint.

^e^Measure completed by participants caring for children under 16 years of age.

^f^Measure completed at 3, 6, 9, and 12 months
