# Supplementary material for: Attitudes About COVID-19 and Health (ATTACH): Online Survey and Mixed Methods Study
Source: JMIR Ment Health. 2021 Oct 7;8(10):e29963. doi: 10.2196/29963 (PMC8500353; doi:10.2196/29963)

**Multimedia Appendix 4** ATTACH study measures

**PHQ-9**

*Over the* ***last 4 weeks*** *how often have you been bothered by any of the following problems?*

|  | | **not at all** | **several days** | **more than half the days** | **nearly every day** |
| --- | --- | --- | --- | --- | --- |
| **1.** | Little interest or pleasure in doing things | *0* | *1* | *2* | *3* |
| **2.** | Feeling down, depressed, or hopeless | *0* | *1* | *2* | *3* |
| **3.** | Trouble falling or staying asleep, or sleeping too much | *0* | *1* | *2* | *3* |
| **4.** | Feeling tired or having little energy | *0* | *1* | *2* | *3* |
| **5.** | Poor appetite or overeating | *0* | *1* | *2* | *3* |
| **6.** | Feeling bad about yourself — or that you are a failure or have let yourself or your family down | *0* | *1* | *2* | *3* |
| **7.** | Trouble concentrating on things, such as reading the newspaper or watching television | *0* | *1* | *2* | *3* |
| **8.** | Moving or speaking so slowly that other people could have noticed? Or the opposite — being so fidgety or restless that you have been moving around a lot more than usual | *0* | *1* | *2* | *3* |
| **9.** | Thoughts that you would be better off dead or of hurting yourself in some way | *0* | *1* | *2* | *3* |
|  | *PHQ-9 total score =* |  | | | |

**UCLA LONELINESS SCALE**

**Instructions:** The following statements describe how people sometimes feel. For each statement, please indicate how often you feel the way described by circling one of the responses below. Here is an example:

Here is an example: How often do you feel happy?

If you never felt happy, you would respond “never”; if you always feel happy, you would respond “always.”

|  | | *Never* | *Rarely* | *Sometimes* | *Often* |
| --- | --- | --- | --- | --- | --- |
| **1.** | I feel in tune with the people around me. | 1 | 2 | 3 | 4 |
| **2.** | I lack companionship. | 1 | 2 | 3 | 4 |
| **3.** | There is no one I can turn to. | 1 | 2 | 3 | 4 |
| **4.** | I do not feel alone. | 1 | 2 | 3 | 4 |
| **5.** | I feel part of a group of friends. | 1 | 2 | 3 | 4 |
| **6.** | I have a lot in common with the people around me. | 1 | 2 | 3 | 4 |
| **7.** | I am no longer close to anyone. | 1 | 2 | 3 | 4 |
| **8.** | My interests and ideas are not shared by those around me. | 1 | 2 | 3 | 4 |
| **9.** | I am an outgoing person. | 1 | 2 | 3 | 4 |
| **10.** | There are people I feel close to. | 1 | 2 | 3 | 4 |
| **11.** | I feel left out. | 1 | 2 | 3 | 4 |
| **12.** | My social relationships are superficial. | 1 | 2 | 3 | 4 |
| **13.** | No one really knows me well. | 1 | 2 | 3 | 4 |
| **14.** | I feel isolated from others. | 1 | 2 | 3 | 4 |
| **14.** | I can find companionship when I want it. | 1 | 2 | 3 | 4 |
| **16.** | There are people who really understand me. | 1 | 2 | 3 | 4 |
| **17.** | I am unhappy being so withdrawn. | 1 | 2 | 3 | 4 |
| **18.** | People are around me but not with me. | 1 | 2 | 3 | 4 |
| **19.** | There are people I can talk to. | 1 | 2 | 3 | 4 |
| **20.** | There are people I can turn to. | 1 | 2 | 3 | 4 |

**PROMIS ANXIETY SCALE**

**
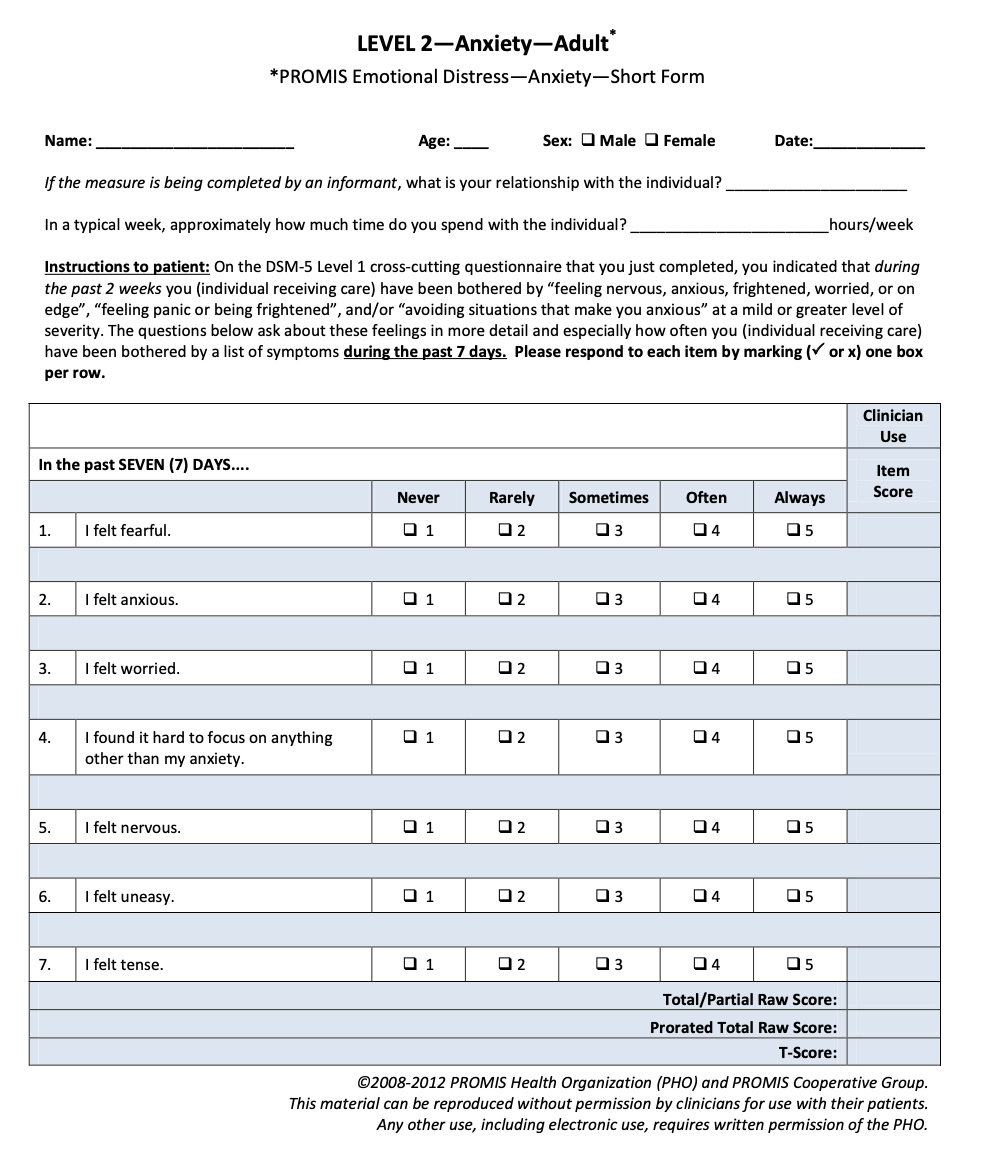
**

**Epidemic – Pandemic Impacts Inventory (EPII)**

Damion J. Grasso, Ph.D.^1^

Margaret J. Briggs-Gowan, Ph.D.^1^

Julian D. Ford, Ph.D., ABPP^1^

Alice S. Carter, Ph.D.^2^

*^1^University of Connecticut School of Medicine*

*^2^University of Massachusetts*

The Epidemic – Pandemic Impacts Inventory (EPII) is a newly developed measure designed to learn about the impact of the coronavirus disease pandemic and future epidemics and pandemics on various domains of personal and family life. Because the EPII is a newly developed measure, there are no psychometric properties, yet available and optimal scoring procedures are not yet determined. Use of the EPII in research studies will help to establish psychometric properties and will likely result in refinement of the assessment tool.

Until further evaluation has been conducted, the EPII should not be used for clinical purposes and is not yet appropriate to inform clinical decision-making or practice.

Researchers are welcome and encouraged to use the EPII in their research studies. Researchers may disseminate the survey using the paper format or may convert items to an online survey format so long as the integrity of the instructions and items is maintained. Please note that users shall not modify items without permission from the developers.

We do ask that you inform the EPII developers of your intention to use the instrument by sending an Email to Dr. Damion Grasso at [dgrasso@uchc.edu](mailto:dgrasso@uchc.edu?subject=Epidemic-Pandemic%20Impacts%20Inventory) with the following information: (1) Principal investigator(s), (2) Purpose of the research study, (3) Population(s) studied, and (4) Study location(s). We appreciate you sharing your findings with us. We are also open and interested in forming collaborative relationships with other investigators.

**EPIDEMIC – PANDEMIC IMPACTS INVENTORY (EPII)**

| **INSTRUCTIONS**  We would like to learn how the coronavirus disease pandemic has changed people's lives. For each statement below, please indicate whether the pandemic has impacted YOU or YOUR FAMILY in the way described.  Check **YES (Me)** if you were impacted.  Check **YES (Person in Home)** if another person (or people) in your home were impacted.  Check **NO** if you and your family were not impacted.  Check **N/A** if the statement does not apply to you or someone in the home.  ****If both* **YES (Me)** *and* **YES (Person in Home)** *are true, check both**** | | | | | |
| --- | --- | --- | --- | --- | --- |
| **Since the coronavirus disease pandemic began, what has changed for you or your family?** | | | | | |
| **PHYSICAL HEALTH PROBLEMS** | | | | |  |
| 50. | Increase in health problems not related to this disease. |  YES (Me)   YES (Person in Home) |  NO |  N/A |  |
| 51. | Less physical activity or exercise. |  YES (Me)   YES (Person in Home) |  NO |  N/A |  |
| 52. | Overeating or eating more unhealthy foods (e.g., junk food). |  YES (Me)   YES (Person in Home) |  NO |  N/A |  |
| 53. | More time sitting down or being sedentary. |  YES (Me)   YES (Person in Home) |  NO |  N/A |  |
| 54. | Important medical procedure cancelled (e.g., surgery). |  YES (Me)   YES (Person in Home) |  NO |  N/A |  |
| 55. | Unable to access medical care for a serious condition (e.g., dialysis, chemotherapy). |  YES (Me)   YES (Person in Home) |  NO |  N/A |  |
| 56. | Got less medical care than usual (e.g., routine or preventive care appointments). |  YES (Me)   YES (Person in Home) |  NO |  N/A |  |
| 57. | Elderly or disabled family member not in the home unable to get the help they need. |  YES (Me)   YES (Person in Home) |  NO |  N/A |  |

**ONLINE SURVEY – at baseline and 10 months**

**WELLBEING**

**GLOBAL HEALTH**


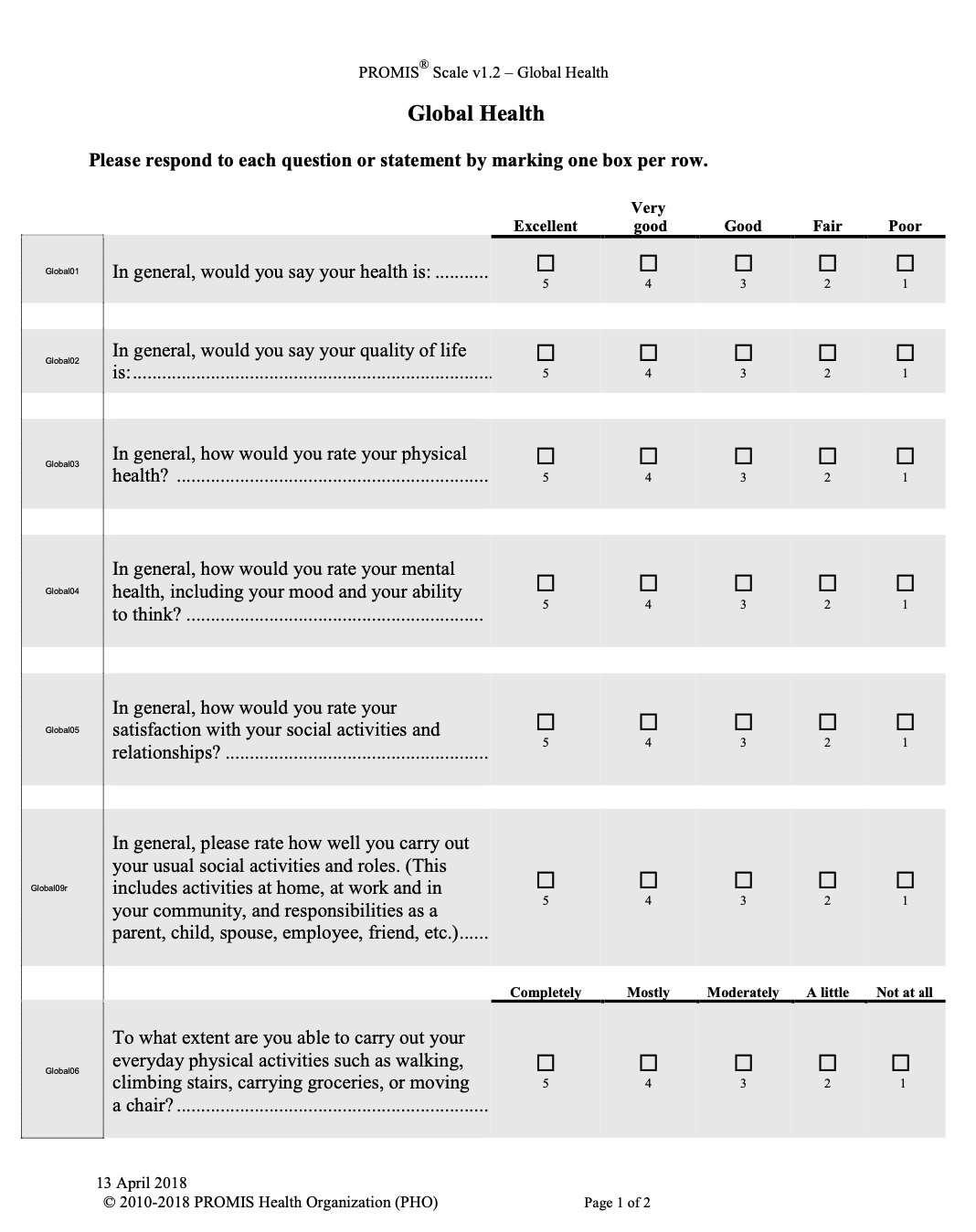

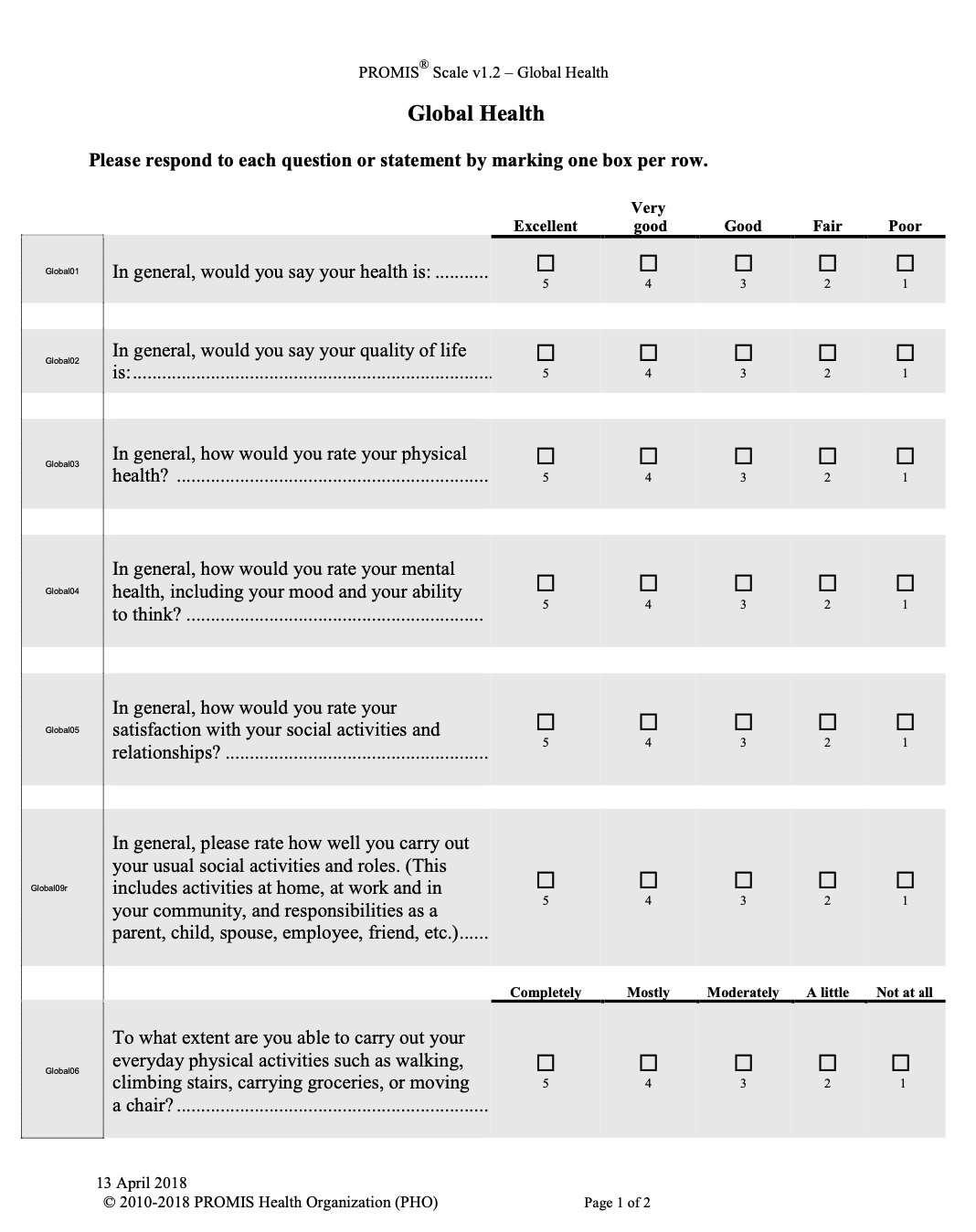

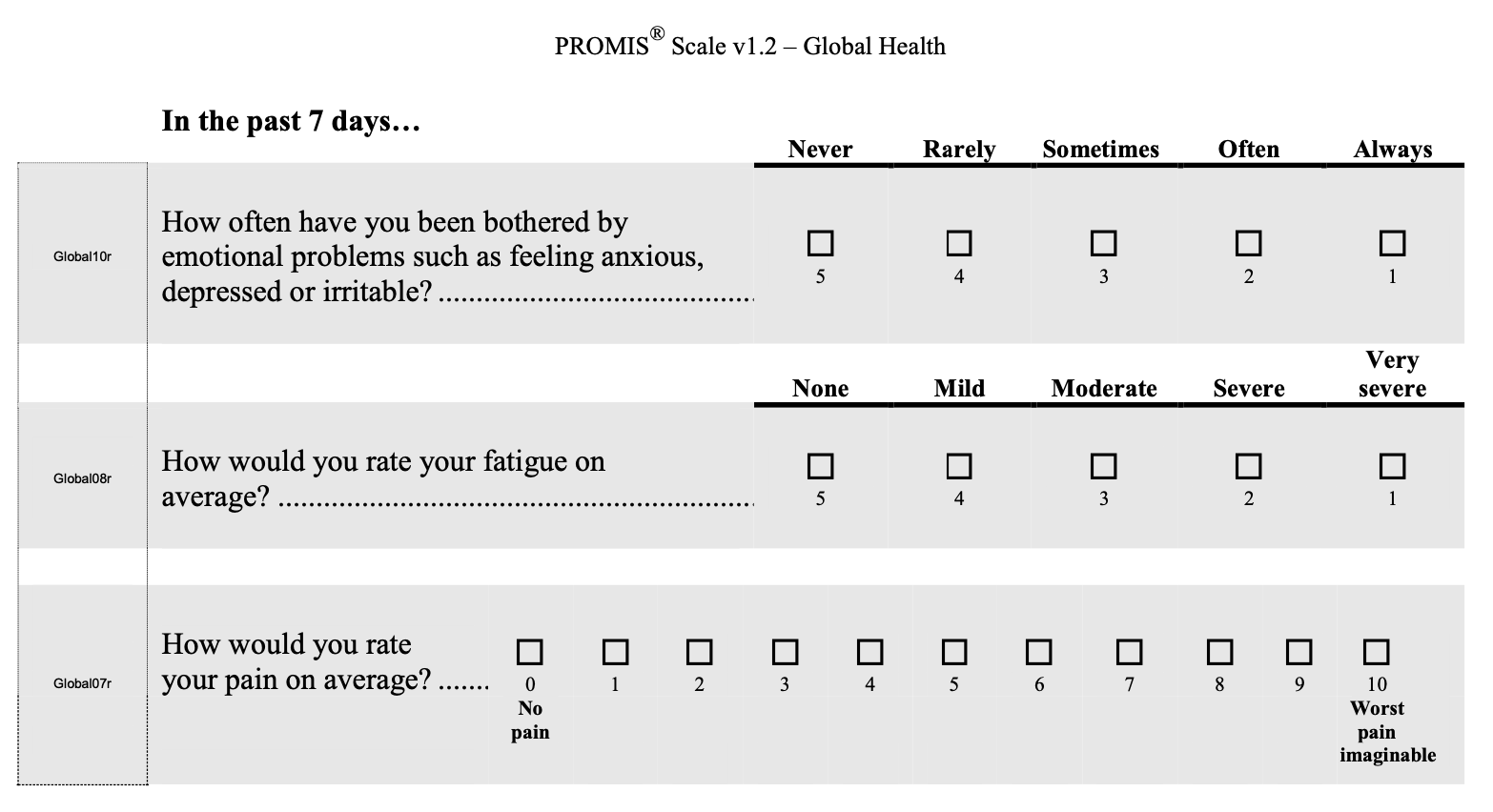

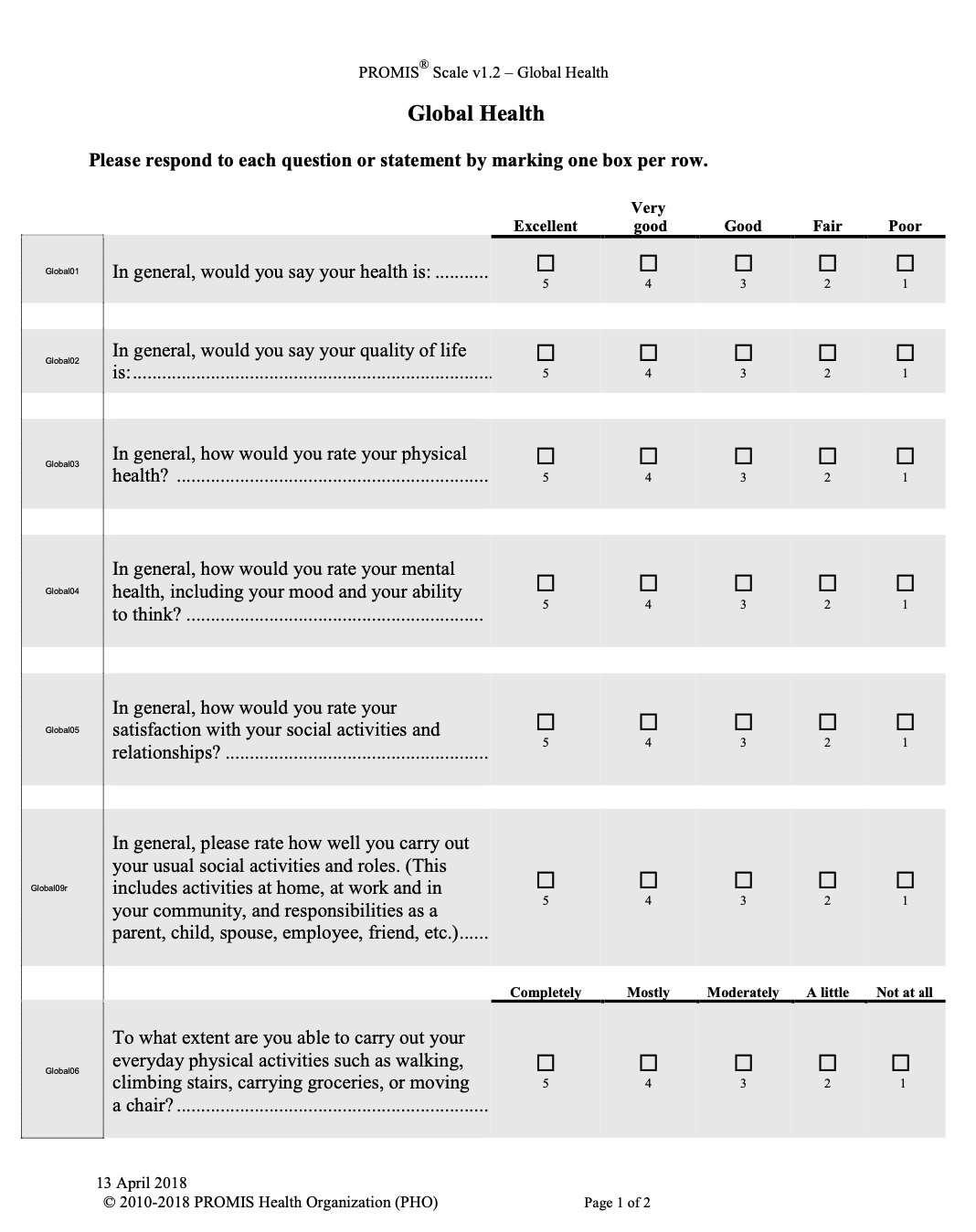

Supplement: Multimedia Appendix 4 [file mental_v8i10e29963_app4.docx]
