# Supplementary material for: Attitudes About COVID-19 and Health (ATTACH): Online Survey and Mixed Methods Study
Source: JMIR Ment Health. 2021 Oct 7;8(10):e29963. doi: 10.2196/29963 (PMC8500353; doi:10.2196/29963)
Supplement: Multimedia Appendix 5 [file mental_v8i10e29963_app5.docx]

**Multimedia Appendix 5** Recruitment strategies and advertisements used in the ATTACH study

## Recruitment

### UK

The research team collaborated by method of consensus to tailor recruitment materials for email and social media posts so that they were appropriate for different audiences, e.g., recruitment materials aimed at university students were differently worded than those for older adults.” Additionally, we co-produced a study logo with undergraduate design students that was used in study materials and featured in a research recruitment video posted on the lab webpages, social media accounts, and YouTube. We aimed to recruit a sample via dissemination through multiple channels. To that end, we used convenience and snowball sampling strategies, social media networks and advertising, involvement from community stakeholders and organizations (see Acknowledgements), research recruitment sites, and university websites and newsletters. Partnerships with charity organizations (see Acknowledgements) were particularly instrumental in aiding recruitment through emails, social media advertising, and by posting the study on their web pages.

We also used paid Facebook advertisements from August 27th, 2020, in which participants could click through to the recruitment webpage where they could enroll. The research team brainstormed ad themes and created them using low-cost photo editing tools. We directed ads to target audiences based on demographic characteristics and user “likes” on Facebook. Ad campaigns were monitored and assessed in real-time through a Facebook Manager dashboard. Based on results (i.e., clicks and study enrollment), the team could either keep an ad active or replace it. Changes were not made on a set schedule but were continuously adapted in line with engagement.

**
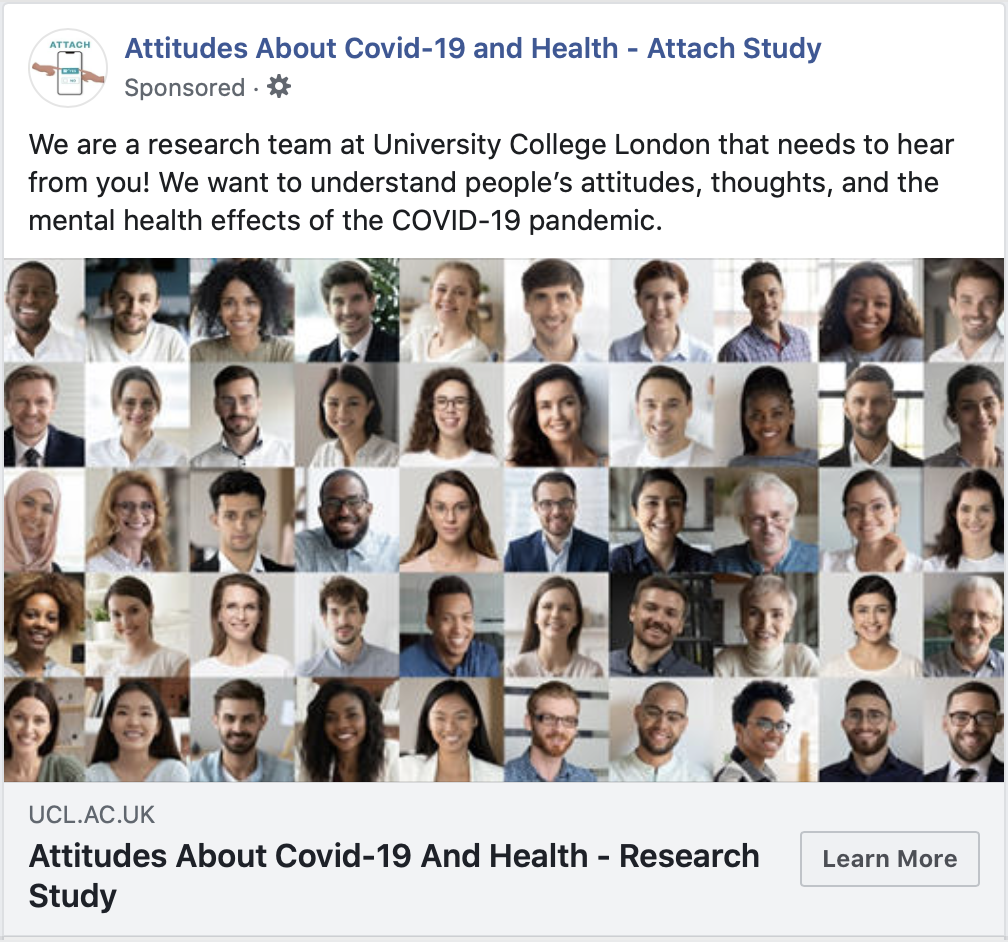
**

### One of the Facebook advertisements used in the ATTACH Study

### USA

We used a multi-pronged approach that included traditional, community-engaged, and contemporary strategies. We engaged with an advisory board of community partners and stakeholders (i.e., caregivers who live in socioeconomically disadvantaged neighborhoods) to develop an electronic study flyer. Verbiage from the flyer also promoted the study on other websites (i.e., All Children Thrive). We utilized community engagement best practices (i.e., cultural brokers, tailored videos, targeted oversampling, and social media snowball techniques). We also recruited adults who had indicated their interest in COVID-19 vaccine trials.

**Mexico**

Researchers distributed study information through Twitter, Facebook, and WhatsApp messages to our research network and colleagues. Paper flyers were posted in supermarkets, convenience stores, and other high traffic areas in two major cities in Mexico, targeting any participants who met study eligibility. Participants were also recruited via local radio stations and national podcasts. University student pools were utilized. Additional recruitment is planned at a psychiatric hospital (pending ethical approval).
