# Supplementary material for: Attitudes About COVID-19 and Health (ATTACH): Online Survey and Mixed Methods Study
Source: JMIR Ment Health. 2021 Oct 7;8(10):e29963. doi: 10.2196/29963 (PMC8500353; doi:10.2196/29963)
Supplement: Multimedia Appendix 6 [file mental_v8i10e29963_app6.docx]

**Multimedia Appendix 6** Mental health disorders and medical conditions reported in the ATTACH study

| **Participant Characteristics** | **N (%)** |
| --- | --- |
|  |  |
| **Mental health disorders** | **N= 242** |
|  |  |
| Depressive disorders | 137 (56.6%) |
| Bipolar and related disorders | 17 (7.0%) |
| Anxiety disorders | 133 (55.0%) |
| Schizophrenia spectrum and other psychotic disorders | 4 (1.7%) |
| Trauma- and stressor-related disorders | 21 (8.7%) |
| Obsessive-compulsive and related disorders | 14 (5.8%) |
| Personality disorders | 5 (2.0%) |
| Feeding and eating disorders | 8 (3.3%) |
| Substance-related and addictive disorders | 2 (0.08%) |
| Dissociative disorders | 2 (0.08%) |
| Neurodevelopmental disorders | 14 (5.8%) |
| Neurocognitive disorders | 9 (3.7%) |
| Somatic symptom and related disorders | 1 (0.04%) |
| Did Not Specify | 3 (0.1%) |
|  |  |
| **Medical conditions** | **N = 402** |
|  |  |
| Anemia | 3 (0.07%) |
| Anorexia Nervosa | 1 (0.02%) |
| Arthritis | 72 (17.9%) |
| Asthma | 134 (33.3%) |
| Autoimmune disease | 6 (1.5%) |
| Cancer | 11 (2.7%) |
| Cerebral or other palsy | 2 (0.05%) |
| Chronic fatigue syndrome | 9 (2.2%) |
| Chronic obstructive pulmonary disease | 11 (2.7%) |
| Deaf or Hearing loss | 2 (0.05%) |
| Diabetes Types 1 and 2 | 61 (15.2%) |
| Epilepsy | 3 (0.07%) |
| Fibromyalgia or chronic pain | 8 (1.9%) |
| Gastrointestinal disorder | 11 (2.7%) |
| Glaucoma | 1 (0.02%) |
| Heart Disease | 22 (5.2%) |
| HIV | 3 (0.07%) |
| Hypertension | 20 (5.0%) |
| Hypothyroidism | 13 (3.2%) |
| Kidney disease | 4 (0.1%) |
| Liver disease | 2 (0.05%) |
| Lung disease | 9 (2.2%) |
| Multiple sclerosis | 2 (0.05%) |
| Neurological disorder | 6 (1.5%) |
| Osteoporosis | 2 (0.05%) |
| Parkinson s disease | 43 (10.7%) |
| Sarcoidosis | 4 (0.1%) |
| Sleep apnea | 5 (1.2%) |
| Sickle cell disease or thalassemia | 2 (0.05%) |
| Splenomegaly | 2 (0.05%) |
| Stenosis | 4 (0.1%) |
| Transplant | 4 (0.1%) |
| Medical other | 13 (3.2%) |
