# Supplementary material for: Attitudes About COVID-19 and Health (ATTACH): Online Survey and Mixed Methods Study
Source: JMIR Ment Health. 2021 Oct 7;8(10):e29963. doi: 10.2196/29963 (PMC8500353; doi:10.2196/29963)
Supplement: Multimedia Appendix 7 [file mental_v8i10e29963_app7.docx]

**Multimedia Appendix 7.** Participant characteristics at baseline in the UK ATTACH Study from June 26 to October 31, 2020

| **Participant Characteristics** | **N (%)** |
| --- | --- |
|  |  |
| **Age** | **1404 (99.9%)** |
|  |  |
| 16-22 | 28 (2.0%) |
| 23-40 | 188 (13.4%) |
| 41-64 | 695 (49.5%) |
| 65-74 | 410 (29.2%) |
| 75+ | 83 (5.9%) |
|  |  |
| **Sex** | **1404 (99.9%)** |
|  |  |
| Female | 1014 (72.2%) |
| Male | 386 (27.5%) |
| Non-binary | 4 (0.3%) |
|  |  |
| **Race** | **1396 (99.3%)** |
|  |  |
| White | 1326 (95.0%) |
| Black | 7 (0.5%) |
| Asian | 28 (2.0%) |
| Mixed/multiple ethnic groups | 17 (1.2%) |
| Arab, Indigenous, LatinX | 4 (0.3%) |
| Did not specify^a^ | 9 (0.6%) |
| Prefer not to say | 5 (0.4%) |
|  |  |
| **First language** | **1282 (91.2%)** |
|  |  |
| English | 1231 (96.0%) |
| Other | 49 (3.8%) |
| Prefer not to say | 2 (0.2%) |
|  |  |
| **Relationship status** | **1282 (91.2%)** |
|  |  |
| In a relationship | 200 (15.6%) |
| Married | 765 (59.7%) |
| Civil partnership | 3 (0.2%) |
| Single | 244 (19.0%) |
| Widowed | 63 (4.9%) |
| Prefer not to say | 7 (0.5%) |
|  |  |
| **Education** | **1282 (91.2%)** |
|  |  |
| Primary school | 1 (0.1%) |
| Secondary school up to 16 years | 102 (8.0%) |
| Higher or secondary or further education (e.g., A-levels, BTEC, etc.) | 180 (14.1%) |
| College or university | 589 (45.9%) |
| Post-graduate degree | 407 (31.7%) |
| Prefer not to say | 3 (0.2%) |
|  |  |
| **Employment status** | **1262 (89.8%)** |
|  |  |
| Employed - travelling to work | 252 (20.0%) |
| Employed - working from home | 253 (20.0%) |
| Furloughed | 21 (1.7%) |
| Sick leave | 6 (0.5%) |
| Unemployed | 32 (2.5%) |
| Disabled - not working | 27 (2.1%) |
| Retired | 540 (42.8%) |
| Homemaker | 24 (1.9%) |
| Student | 32 (2.5%) |
| Self-employed | 36 (2.9%) |
| Other (e.g., carer, casual worker/freelance, parental leave, volunteer) | 39 (3.1%) |
|  |  |
| **Key/essential worker status** | **1377 (98.0%)** |
|  |  |
| Yes | 277 (20.1%) |
| No | 1098 (79.7%) |
| Prefer not to say | 2 (0.1%) |
|  |  |
| **Number of people living in the household** | **1282 (91.2%)** |
|  |  |
| 1 | 257 (20.0%) |
| 2 | 681 (53.1%) |
| 3 | 183 (14.3%) |
| 4 | 119 (9.3%) |
| 5 or more | 37 (2.9%) |
| Prefer not to say | 5 (0.4%) |
|  |  |
| **Caregiver of child/children under 16 years** | **1282 (91.2%)** |
|  |  |
| No | 1117 (87.1%) |
| Yes | 139 (10.8%) |
| Did not provide a response | 24 (1.9%) |
| Prefer not to say | 2 (0.2%) |
|  |  |
| **Mental health disorders** | **1282 (91.2%)** |
|  |  |
| No | 1017 (79.3%) |
| Yes | 242 (18.9%) |
| Prefer not to say | 23 (1.8%) |
|  |  |
| **Medical conditions** | **1282 (91.2%)** |
|  |  |
| No | 880 (68.6%) |
| Yes | 402 (31.4%) |
|  |  |
| **Household income affected by COVID-19** | **1259 (89.6%)** |
|  |  |
| No | 947 (75.2%) |
| Yes | 300 (23.8%) |
| Prefer not to say | 12 (1.0%) |
|  |  |
| **Country of residence** | **1058 (75.3)** |
|  |  |
| England | 938 |
| Scotland | 86 |
| Wales | 25 |
| Northern Ireland | 8 |
| Jersey | 1 |
|  |  |
| **Index of Multiple Deprivation (IMD) quintile (1 = most deprived)^b^** | **1028 (73.2%)** |
|  |  |
| 1 | 83 (8.1%) |
| 2 | 161 (15.7%) |
| 3 | 246 (23.9%) |
| 4 | 291 (28.3%) |
| 5 | 247 (24.0%) |
|  |  |
| **Population density** | **1032 (73.5%)** |
|  |  |
| Mean ± SD | 28.1 ± 35.2 |
| Min, Max | 0.0, 264.6 |
|  |  |
| **Political views (0 = left, 100 = right)** | **1153 (82.1%)** |
|  |  |
| Mean ± SD | 42.3 ± 22.2 |
| Min, Max | .00, 100 |

^a^Participants only provided race on the app but not the survey where there were not multiple options were not available.

^b^Combined using IMD quintile scores from England (2019), Northern Ireland (2017), Scotland (2020), and Wales (2019).
