# Supplementary material for: Attitudes About COVID-19 and Health (ATTACH): Online Survey and Mixed Methods Study
Source: JMIR Ment Health. 2021 Oct 7;8(10):e29963. doi: 10.2196/29963 (PMC8500353; doi:10.2196/29963)
Supplement: Multimedia Appendix 8 [file mental_v8i10e29963_app8.docx]

**Multimedia Appendix 8.** Participant clinical classifications and severity on self-reported outcomes at baseline in the UK ATTACH study from June 26 to October 31, 2020

| **Self-reported Outcomes** | **N (%)** |
| --- | --- |
|  |  |
| **Anxiety: PROMIS 7a^a^** | **1244 (89%)** |
|  |  |
| None to Slight | 627 (50.4%) |
| Mild | 265 (21.3%) |
| Moderate | 294 (23.6%) |
| Severe | 58 (4.7%) |
|  |  |
| **Depression PHQ-9^b^** | **1217 (86.6%)** |
|  |  |
| Minimal | 695 (57.1%) |
| Mild | 289 (23.7%) |
| Moderate | 124 (10.2%) |
| Moderately Severe | 63 (5.2%) |
| Severe | 46 (3.8%) |
|  |  |
| **Social Isolation: UCLA^c^ 3-item** | **1232 (87.7%)** |
|  |  |
| Hardly Ever or Never Lonely | 247 (20.0%) |
| Lonely Some of the Time | 529 (42.9%) |
| Often Lonely | 456 (37.1%) |
|  |  |
| **Physical health: PROMIS** | **1208 (85.9%)** |
|  |  |
| Excellent | 84 (7.0%) |
| Very Good | 346 (28.6%) |
| Good | 530 (43.9%) |
| Fair | 193 (16.0%) |
| Poor | 55 (4.6%) |
|  |  |
| **Quality of Life: PROMIS** | **1206 (85.8%)** |
|  |  |
| Excellent | 135 (11.2%) |
| Very Good | 199 (16.5%) |
| Good | 396 (32.8%) |
| Fair | 293 (24.3%) |
| Poor | 183 (15.2%) |

^a^ PROMIS: Patient-Reported Outcomes Measurement Information System

^b^ PHQ-9: Patient Health Questionnaire

^c^ UCLA = UCLA Loneliness Scale
