# Supplementary material for: Attitudes About COVID-19 and Health (ATTACH): Online Survey and Mixed Methods Study
Source: JMIR Ment Health. 2021 Oct 7;8(10):e29963. doi: 10.2196/29963 (PMC8500353; doi:10.2196/29963)
Supplement: Multimedia Appendix 9 [file mental_v8i10e29963_app9.docx]

**Multimedia Appendix 9.** Participant characteristics at baseline in the USA ATTACH Study from June 26 to October 31, 2020

| **Participant Characteristics (n = 90)** | **N (%)** |
| --- | --- |
|  |  |
| **Age** |  |
|  |  |
| 16-22 | 1 (0.2%) |
| 23-40 | 31 (34.4%) |
| 41-64 | 49 (54.4%) |
| 65-74 | 9 (10.0%) |
|  |  |
| **Sex** |  |
|  |  |
| Female | 68 (75.6%) |
| Male | 22 (24.4%) |
|  |  |
| **Race** |  |
|  |  |
| White | 31 (34.4%) |
| Black | 45 (50.0%) |
| Hispanic/LatinX | 8 (8.9%) |
| Asian | 1 (1.1%) |
| American Indian or Alaska Native | 1 (1.1%) |
| Mixed/multiple ethnic groups | 4 (4.4%) |
|  |  |
| **First language** |  |
|  |  |
| English | 78 (86.7%) |
| Spanish | 11 (12.2%) |
| Portuguese | 1 (1.1%) |
|  |  |
| **Relationship status** |  |
|  |  |
| In a relationship | 16 (17.8%) |
| Married | 42 (46.7%) |
| Single | 31 (34.4%) |
| Widowed | 1 (1.1%) |
|  |  |
| **Education** |  |
|  |  |
| 9th-12th grade or less | 1 (1.1%) |
| High School Graduate | 7 (7.8%) |
| Some college or certification | 18 (20%) |
| College graduate | 30 (33.3%) |
| Post-graduate degree | 34 (37.8%) |
| Prefer not to say | 3 (0.2%) |
|  |  |
| **Employment status** |  |
|  |  |
| Employed - travelling to work | 33 (36.7%) |
| Employed - working from home | 35 (38.9%) |
| Employed - mix of work and home | 1 (1.1%) |
| Furloughed - without pay | 2 (2.2%) |
| Part-time | 1 (1.1%) |
| Sick leave | 2 (2.2%) |
| Disabled | 2 (2.2%) |
| Retired | 3 (3.3%) |
| Homemaker | 3 (3.3%) |
| Unemployed | 7 (7.8%) |
| Prefer not to say | 1 (1.1%) |
|  |  |
| **Keyworker status** |  |
|  |  |
| Yes | 40 (44.4%) |
| No | 50 (55.6%) |
|  |  |
| **Number of people living in household** |  |
|  |  |
| 1 | 18 (20%) |
| 2 | 33 (36.7%) |
| 3 | 10 (11.1%) |
| 4 | 16 (17.8%) |
| 5 or more | 13 (14.4%) |
|  |  |
| **Caregiver of child/children under 16 years** |  |
|  |  |
| No | 56 (62.2%) |
| Yes | 34 (37.8%) |
|  |  |
| **Mental health disorders** |  |
|  |  |
| No | 77 (85.6%) |
| Yes | 12 (13.3%) |
| Prefer not to say | 1 (1.1%) |
|  |  |
| **Medical conditions** |  |
|  |  |
| No | 63 (70.0%) |
| Yes | 26 (28.9%) |
| Prefer not to say | 1 (1.1%) |
|  |  |
| **Household income affected by COVID-19** |  |
|  |  |
| No | 68 (75.6%) |
| Yes | 22 (24.4%) |
|  |  |
| **Political views (0 = left, 100 = right)** |  |
|  |  |
| Mean ± SD | 34.0 ± 21.6 |
| Min, Max | .00, 100 |
