# Supplementary material for: Attitudes About COVID-19 and Health (ATTACH): Online Survey and Mixed Methods Study
Source: JMIR Ment Health. 2021 Oct 7;8(10):e29963. doi: 10.2196/29963 (PMC8500353; doi:10.2196/29963)
Supplement: Multimedia Appendix 10 [file mental_v8i10e29963_app10.docx]

**Multimedia Appendix 10.** Participant characteristics at baseline in the Mexico ATTACH Study from October 6 to October 31, 2020

| **Participant Characteristics (n = 80)** | **N (%)** |
| --- | --- |
|  |  |
| **Age** |  |
|  |  |
| 16-22 | 29 (36.2%) |
| 23-40 | 42 (52.5%) |
| 41-64 | 6 (7.5%) |
| 65-74 | 3 (3.7%) |
|  |  |
| **Sex** |  |
|  |  |
| Female | 59 (73.8%) |
| Male  Non-binary | 19 (23.8%)  2 (2.5%) |
|  |  |
| **Race** |  |
|  |  |
| Mexican | 23 (28.7%) |
| Mixed/Multiple Ethnic Groups | 40 (50%) |
| White | 10 (12.5%) |
| Asian | 1 (1.25%) |
| Indigenous | 1 (2.25%) |
| Did not specify | 1 (1.25%) |
| No response | 4 (5%) |
|  |  |
| **First language** |  |
|  |  |
| Spanish | 79 (98.8%) |
| Did not specify | 1 (1.25%) |
|  |  |
| **Relationship status** |  |
|  |  |
| In a relationship | 8 (10%) |
| Married | 20 (25%) |
| Single | 48 (60%) |
| Divorced | 3 (3.75%) |
| Widowed | 1 (1.25%) |
|  |  |
| **Education** |  |
|  |  |
| High School Graduate (12 years) | 32 (40%) |
| College graduate (16-17 years) | 28 (35%) |
| Post-graduate degree (19-20 years) | 9 (11.2%) |
| Post-graduate degree (PhD, 21-25 years) | 9 (11.2%) |
| Other | 2 (2.5%) |
|  |  |
| **Employment status** |  |
|  |  |
| Employed – with economic income | 38 (47.5%) |
| Employed - without economic income (i.e., furloughed) | 6 (7.5%) |
| Unemployed – with economic income from informal activities | 6 (7.5%) |
| Unemployed - without pay | 30 (37.5%) |
|  |  |
| **Keyworker or essential status** |  |
|  |  |
| Yes | 13 (16.2%) |
| No | 26 (32.5%) |
| No response | 41(51.2%) |
|  |  |
| **Number of people living in household** |  |
|  |  |
| 1 | 7 (8.75%) |
| 2 | 15 (18.8%) |
| 3 | 17 (21.2%) |
| 4 | 20 (25%) |
| 5 or more | 21 (26.2%) |
|  |  |
| **Caregiver of child/children under 16 years** |  |
|  |  |
| No | 62 (77.5%) |
| Yes | 17 (21.%) |
| Prefer not to say | 1 (1.25%) |
|  |  |
| **Mental health disorders** |  |
|  |  |
| No | 54 (67.5%) |
| Yes | 22 (27.5%) |
| Prefer not to say | 4 (5%) |
|  |  |
| **Household income affected by COVID-19** |  |
|  |  |
| Yes | 32 (40.4%) |
| No | 47 (58.8%) |
| It could be if I followed the confinement measures | 1 (1.25%) |
|  |  |
| **Political views (0 = left, 100 = right)** |  |
|  |  |
| Mean ± SD | 46.87 ± 25.3 |
| Min, Max | .00, 100 |
